# Enhancing Natural Killer and CD8^+^ T Cell-Mediated Anticancer Cytotoxicity and Proliferation of CD8^+^ T Cells with HLA-E Monospecific Monoclonal Antibodies

**DOI:** 10.1089/mab.2018.0043

**Published:** 2019-04-22

**Authors:** Mepur H. Ravindranath, Edward J Filippone, Asokan Devarajan, Shahab Asgharzadeh

**Affiliations:** ^1^ Department of Hematology and Oncology, Children's Hospital, Los Angeles, California.; ^2^ Division of Nephrology, Department of Medicine, Sidney Kimmel Medical College, Thomas Jefferson University, Philadelphia, Pennsylvania.; ^3^ Division of Cardiology, Department of Medicine, David Geffen School of Medicine, University of California Los Angeles, Los Angeles, California.; ^4^ Department of Pediatrics and Pathology, Children's Hospital, Keck School of Medicine, USC, Los Angeles, California.

**Keywords:** human leukocyte antigen, monospecific versus polyreactive, monoclonal antibody, natural killer receptors, single antigen bead assay, mean fluorescent intensity

## Abstract

Cytotoxic NK/CD8^+^ T cells interact with MHC-I ligands on tumor cells through either activating or inhibiting receptors. One of the inhibitory receptors is CD94/NKG2A. The NK/CD8^+^ T cell cytotoxic capability is lost when tumor-associated human leukocyte antigen, HLA-E, binds the CD94/NKG2A receptor, resulting in tumor progression and reduced survival. Failure of cancer patients to respond to natural killer (NK) cell therapies could be due to HLA-E overexpression in tumor tissues. Preventing the inhibitory receptor–ligand interaction by either receptor- or ligand-specific monoclonal antibodies (mAbs) is an innovative passive immunotherapeutic strategy for cancer. Since receptors and ligands can be monomeric or homo- or heterodimeric proteins, the efficacy of mAbs may rely on their ability to distinguish monospecific (private) functional epitopes from nonfunctional common (public) epitopes. We developed monospecific anti-HLA-E mAbs (e.g., TFL-033) that recognize only HLA-E-specific epitopes, but not epitopes shared with other HLA class-I loci as occurs with currently available polyreactive anti-HLA-E mAbs. Interestingly the amino acid sequences in the α1 and α2 helices of HLA-E, critical for the recognition of the mAb TFL-033, are strikingly the same sequences recognized by the CD94/NKG2A inhibitory receptors on NK/CD8^+^ cells. Such monospecific mAbs can block the CD94/NKG2A interaction with HLA-E to restore NK cell and CD8^+^ anticancer cell cytotoxicity. Furthermore, the HLA-E monospecific mAbs significantly promoted the proliferation of the CD4^−^/CD8^+^ T cells. These monospecific mAbs are also invaluable for the specific demonstration of HLA-E on tumor biopsies, potentially indicating those tumors most likely to respond to such therapy. Thus, they can be used to enhance passive immunotherapy once phased preclinical studies and clinical trials are completed. On principle, we postulate that NK cell passive immunotherapy should capitalize on both of these features of monospecific HLA-E mAbs, that is, the specific determination HLA-E expression on a particular tumor and the enhancement of NK cell/CD8^+^ cytotoxicity if HLA-E positive.

## Introduction

Active specific and passive immunotherapies of human cancers aim to reprogram the metabolic profile of the tumors and their microenvironment during the progression of cancers. There is a need to identify and detect the specific biomarkers of the metabolic profile of a tumor or an immune cell on an individual patient basis. Biomarkers, whether composed of proteins or polysaccharides or lipids, can be monomeric, homodimeric, or heterodimeric. Monoclonal antibodies (mAbs) are indeed the most valuable tools for their specific immunodiagnosis. Furthermore, biomarkers may contain common (public or shared) epitopes in addition to specific and individual or unique epitopes. Therefore, it is critical to examine whether a mAb is genuinely monospecific for a biomarker, by demonstrating reaction only with private epitopes as opposed to shared epitopes.

Although cellular immunotherapy has successfully explored antitumor CD8^+^ T cell responses, it is increasingly realized that human cancers reprogram by developing strategies to escape T cell recognition. Counter strategies are needed to upregulate killer T cells as well as manipulate natural killer (NK) cells to attack the cancer cells. The NK cells share expression of cell surface antigens and effector molecules, such as CD2, CD7, CD90, perforin, granzyme A, and interferon-γ, with T lymphocytes on one hand, and the surface antigens such as CD11b and CD11c with myeloid cells on the other, based on specific genes required for T cell receptor (TCR) rearrangement.^(1)^ The 112 genes for cell surface and signaling receptors and molecules are found to be significantly upregulated in NK cells,^(2)^ and such upregulation may exhibit both inter- and intraindividual variations during various stages of tumorigenesis, metastasis, and infections. Therefore, personalized therapy should precisely monitor these variations in the molecular expression of such biomarkers. Indeed, precise monitoring requires well-defined and precise tools.

The NK cell receptors of the immunoglobulin (Ig) superfamily (e.g., killer cell Ig-like receptors, also known as KIRs and Ig-like transcripts) recognize human major histocompatibility complex (MHC) class-I molecules (human leukocyte antigens [HLAs] -Ia, -Ib, and MICA/MICB).^(3–6)^ Some of these receptors transmit activating signals, whereas others mediate inhibition. One major class of genes controlling both activating and inhibitory NK cell receptor groups (NKGs) is *Klrc1* [NKG2A], *Klrc2* [NKG2C], and *Klrk1* [NKG2D] and are involved in specific interactions with the MHC of tumor cells and virally infected cells. Each NKG2 subunit is a type II glycoprotein belonging to the C-type lectin superfamily, with an extracellular domain with transmembrane and cytoplasmic segments. The analogous murine Ly49 family receptors^(5)^ and human KIR^(3,4)^ both bind to cell surface HLA-Ia molecules on target cells. Natural killer cell group 2 (NKG2) receptors interact specifically with HLA-Ib and MICA/MICB as the homodimer (NKG2D) or as heterodimers (NKG2A, NKG2C), and such interactions are highly conserved across species. Most of the NKG2 isoforms (NKG2A, B, C, E, and H, but not NKG2D) form disulfide-linked heterodimers with an invariant chain, CD94.^(7,8)^ NKG2A and NKG2B, which are alternatively spliced products from a single gene, have two immunoreceptor tyrosine-based inhibitory motifs in their cytoplasmic domains and form inhibitory receptors when complexed with CD94.^(2,7)^ The specific ligands for CD94/NKG2A/C heterodimers are identified as nonclassical class-I molecules (HLA-Ib) in humans.^(8–10)^

### The homodimer NKG2D interacts with MICA on tumor cell surface

NKG2D is a unique activating receptor of NK cells that share little similarity with the other isoforms of NKG2 receptors and does not associate with CD94. The receptor is on natural killer T cells (NKT) cells, subsets of γδ T cells,^(2)^ activated macrophages,^(11)^ and naive human CD8^+^ T cells. CD4^+^T cells can be induced to express it under certain pathological conditions, such as Crohn's disease, juvenile-onset lupus, and cytomegalovirus infection.^(12)^ NKG2D recognizes highly polymorphic MHC loci MICA (60 allelic variants) and MICB (30 allelic variants), also known as “stress-induced MHC” on normal cells,^(13,14)^ tumor,^(15)^ and virally infected cells.^(16)^ NKG2D–MICA interaction leads to tumor cell destruction, mediated by the release of perforin by the NK and other immune cells.^(11)^ NKG2D is also to recognize other protein ligands, encoded by genes that encode functional proteins [in square brackets] in humans (RAET1E [ULBP4], RAET1G [ULBP5], RAET1H [ULBP2], RAET1I [ULBP1], RAET1L [ULBP6], and RAET1N [ULBP3]).^(17)^

The interaction of the activating NK cell receptor NKG2D with tumor cell surface MICA and MICB has been extensively studied^(18–29)^ in gastrointestinal (GI) epithelium and several epithelial tumors. The extracellular transport of MICA and MICB is independent of the general peptide processing machinery that is required for the assembly of peptides for other antigen-presenting HLA-I molecules.^(13–18)^ The MICA/B proteins share ∼30% identical amino acid residues throughout α1, α2, and α3 domains with other HLA class-I proteins. They possess seven to eight N-linked glycosylation sites, unique transmembrane and cytoplasmic tail sequences, and three extra cysteine residues in the α1 and α3 domains. Unlike HLA class-I proteins, they do not dimerize with β2-microglobulin (β2m).

Tumor cells may escape NKG2D–MICA-mediated immune attack by disulfide–isomerase-enabled proteolytic degrading and shedding of MICA.^(18)^ Ferrari de Andrade et al.^(19)^ have designed antibodies targeting the MICA α3 domain, the site of proteolytic cleavage for shedding, and found that these antibodies prevented loss of cell surface MICA and MICB in human cancer cells. Interestingly, these antibodies do not inhibit binding of the α1 and α2 domains with receptors and allowed continued NKG2D–MICA interactions to inhibit murine tumor progression in mouse models and reduced human melanoma metastases in a humanized mouse model. However, in some cancers at the advanced stage, the expression of ligands for NKG2D may promote tumor progression rather than regression. A recent report documents NKG2D activation-mediated tumor progression in a model of inflammation-driven liver cancer,^(20)^ with the critical differentiating factor purported to be the surrounding inflammation. Hence, the nature of the microenvironment within and surrounding tumors may have the potential of altering the impact of NKG2D activation, and this aspect deserves attention while studying the impact of NKG2D interaction with MICA and other such ligands on the tumor cell surface.

In recent years, NKG2D–MICA ligand interactions in different cancers have been reviewed extensively.^(21–25)^ The crystal structure of MICA shows restructured α1 and α2 helices, the regions interacting with NKG2D, and there is altered folding with a shallow remnant of a peptide-binding groove without a peptide.^(26–28)^ The interaction between deglycosylated recombinant MICA and NKG2D shows that the NKG2D can bind to the presumptive peptide grooves of α1 and α2 domains at the top of the MICA platform, analogous to αβTCR recognition of MHC class-I proteins.^(29)^ Since there are 60 allelic variants of MICA proteins, remarkable variations may occur in the α1 and α2 domains affecting binding of NKG2D with the MICA alleles.^(30)^ However, it is also suggested that the NKG2D binding sites are on the underside of the MICA α1 and α2 domains, this region is comparable with the cryptic domain in classical and nonclassical HLA-I proteins masked by β2m. This domain in classical and nonclassical HLA-I loci contains mostly shared or public epitopes.^(31)^

## Cancer-Associated HLA Class-I Molecules and NKG2A/CD94

As noted previously, NKG2 receptors different from NKG2D may mediate NK suppression of tumor killing rather than activation. Earlier studies reported that NK cells can kill autologous and allogeneic tumor cells without involving the MHC.^(32)^ Lymphomas deficient in MHC antigens were rejected *in vivo* in contrast to MHC-expressing lymphomas.^(33)^ Such loss of MHC expression failed to activate inhibitory NKG2 receptors (other than the activating NKG2D).

Similarly, Liao et al.^(34)^ observed that the normal T cell blasts from MHC-I-deficient mutant mice are effectively targeted by NK cells *in vitro*, a finding that supports the speculation that HLA class-I expressed on regular T cell blasts may result in inhibition of NK-mediated cell killing. Furthermore, an inverse correlation was noted between *in vitro* NK-mediated cytolysis and the level of target cell HLA class-I expression.^(35)^ However, no such correlation was noticed with target cell HLA class-II molecules.^(35)^

Moretta et al.^(36)^ have studied the interaction between the CD94 receptor and HLA class-I. The cells expressed HLA-B7 protected target cells from NK lysis. The protection was abolished by CD94-specific mAbs (XA-185) but not by the mAbs conjugated with the soluble CD94 molecule. The CD94-specific mAbs (XA-185) did not react with the inhibitory NKG2A receptor, as it failed to stain COS-7 cell lines (fibroblast-like cell lines) transfected only with NKG2A but stained those transfected with both CD94 and NKG2A. In contrast, yet another mAb Z199, which does not recognize CD94, strongly reacted with COS cells cotransfected with CD94/NKG2A,^(37)^ suggesting the occurrence of heterodimerization of NKG2A with CD94. Contrastingly, the same group documented the inhibitory role of CD94/NKG2A in a predominantly CD8^+^ fraction of human T lymphocytes.^(38)^ In the presence of IL-15, the CD8^+^ T cells expressed CD94 *de novo*. CD94 expression commenced 4–6 days after addition of IL-15. Both CD4^+^ and CD8^+^ cells expressed CD94, but the simultaneous expression of NKG2A occurred only with CD8^+^ cells. Similar data were obtained in T cell populations activated in mixed lymphocyte cultures in the presence of IL-15. The expression of CD94/NKG2A diminished the allospecific cytolytic activity by mixed lymphocyte culture-derived T cell populations. Importantly, the cytolysis was reinstated by adding anti-CD94 mAbs, which masks the inhibitory NK cell CD94. These and similar observations^(39)^ confirmed that the binding of CD94/NKG2A with HLA class-I on tumor cells protected them from cytotoxic killing by both NK cells and a subset of CD8^+^ T cells, although the precise mechanism remained to be clarified.

## Tumor-Associated HLA-E as the Specific Ligand for CD94/NKG2A

**FIG. 1.**
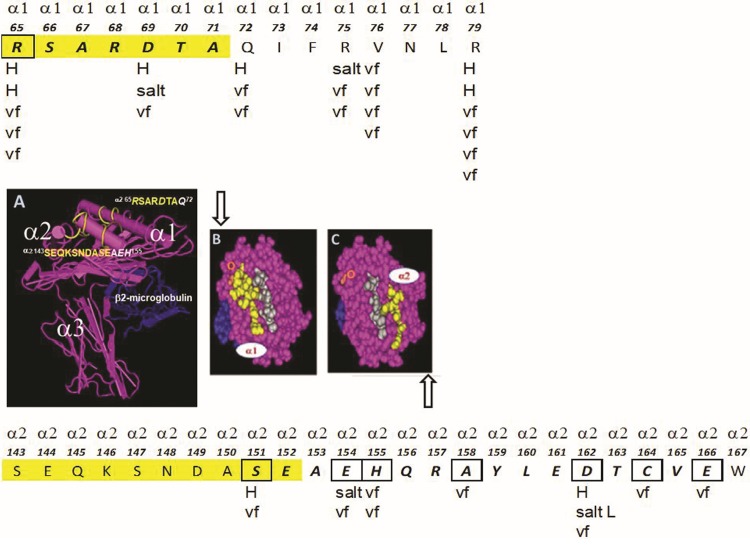
The structure of the nonclassical (HLA class-Ib) human leukocyte antigen HLA-E. **(A)** The structural orientation of the three α-helices of the heavy chain with β2m is illustrated. The peptide in the groove is not shown for the purpose of indicating the location of the specific amino acid sequences or epitopes on the α1 and α2 helices, which serve as the ligand for monospecific anti-HLA-E mAbs and CD94 and NKG2a receptors on the cell surface of NK cells and CD8^+^ T cells. The sequences in yellow in **(A–C)** are the specific sites of the ligand for mAb TFL-033, assessed based on dosimetric peptide inhibition studies. **(A)** The structure of intact or native HLA-E showing epitopes (the specific amino acid sequences) that inhibit the binding of mAb TFL-033 to HLA-E coated on the beads. The sequences RSARDTA at α1 and SEQKSNDASE at α2 are the TFL-033 binding regions established based on the peptide-inhibition study.^(54)^ **(B, C)** The structure of the amino acids in the α1 and α2 helices that are recognized by CD94 and NKG2A. The letters H, Salt, and vf refer to the sites of hydrogen bonding, salt linkages, and van der Wall forces between CD94 and NG2A with the α1 and α2 helices, respectively. Gray of amino acid sequences refers to the sequences of the leader peptide sequence attached to the groove. The attachment of the peptide on the groove renders stability to dimerization of HLA-E heavy chains and β2m. Theoretically if a mAb binds anywhere in the yellow regions on the α1 and α2 helices, it would block the binding of the inhibitory receptor of NK cells. This is the basis for the hypothesis that monospecific anti-HLA-E mAbs define the potential of the mAb to avert the interaction between inhibitory receptors and HLA-E, and thereby preventing the “inaction” or “switching off” of the NK cell function that would otherwise enable tumor cell escape. The boxes refer to the exact amino acids in HLA-E sequences that interact or bind with the amino acids of CD94 and NKG2A. The linkgage types are shown in letters below the boxes. β2m, β2-microglobulin; HLA, human leukocyte antigen; mAb, monoclonal antibody; NK, natural killer; NKG2, natural killer cell group 2.

HLA class-I molecules include highly polymorphic classical HLA class-Ia (HLA-A, -B, and -C alleles) and the least polymorphic nonclassical HLA class-Ib (HLA-E, -F, or -G). Literature increasingly documents that HLA-E may function as the specific ligand for the inhibitory NKG2 receptors. The fundamental structure of HLA-E is not much different from any one of the HLA class-Ia molecules (Fig. 1A). Interestingly, the HLA-E heavy chain shares several amino acid sequences with HLA class-Ia, despite several unique differences in the amino acid sequences or epitopes between HLA-E and HLA-Ia alleles (Tables 1 and 2). HLA-E functions by assembling with the leader peptide of HLA class-Ia/-Ib proteins as its cognate peptide.^(40)^ The leader peptide is loaded at the N terminus of the newly synthesized HLA class-I molecule. The role of leader peptide is to ensure translocation of the newly synthesized HLA class-I heavy chain into the endoplasmic reticulum (ER) and promote the transportation of the HLA across the ER. Upon completion of the task, the leader peptide is cleaved from the heavy chain of HLA by a signal peptidase^(41)^ and enters the cytoplasm. From there, the leader peptide itself must be transported across the ER membrane, which requires regular transporter associated with antigen processing (TAP) expression and function. Normal maturation and insertion of HLA-E into the cell surface membrane requires the leader peptide of HLA class-I molecules or leader peptides of HLA-F or HLA-G (but not the leader peptide of HLA-E itself) to bind the HLA-E peptide grove in the ER. A newly synthesized HLA-E matures and forms a stable complex only in the presence of the leader peptide of most HLA-A, -B, -C, and -G molecules, but not its own leader peptide.^(40)^

**Table 1.** Gene Sequence-Based (First Row of Numbers) and Secreted Heavy Chain-Based Amino Acid Sequences (Second Row of Numbers) of Nonclassical HLA-Ib, HLA-E Showing Private (Monospecific) and Public (Polyspecific) Epitopes in Boxes

**Table 2.** Amino Acid Sequences (Epitopes) Shared with Classical HLA-Ia Loci (HLA-A, HLA-B, HAL-Cw, and Nonclassical HLA-Ib Loci (HLA-F and HLA-G)

*HLA-E peptide sequences*	*No. of amino acids*	*HLA-I alleles*					*Specificity*
		*Classical Ia*			*Nonclassical Ib*		
		*A*	*B*	*Cw*	*F*	*G*	
^47^PRAPWMEQE^55^	9	1	0	0	0	0	A^*^3306
^59^EYWDRETR^65^	8	5	0	0	0	0	A-restricted
^65^RSARDTA^71^ (^*^)	***6***	***0***	***0***	***0***	***0***	***0***	***E-restricted***
^90^AGSHTLQW^97^	8	1	10	48	0	0	Polyspecific
^108^RFLRGYE^123^	7	24	0	0	0	0	A-restricted
^115^QFAYDGKDY^123^	9	1	194	75	0	0	Polyspecific
^117^AYDGKDY^123^	7	491	831	271	21	30	Polyspecific
^126^LNEDLRSWTA^135^	10	239	210	261	21	30	Polyspecific
^137^DTAAQI^142^	6	0	824	248	0	30	Polyspecific
^137^DTAAQIS^143^	7	0	52	4	0	30	Polyspecific
^143^SEQKSNDASE^152^(^*^)	***10***	***0***	***0***	***0***	***0***	***0***	***E-restricted***
^157^RAYLED^162^	6	0	1	0	0	0	B^*^8201
^163^TCVEWL^168^	6	282	206	200	0	30	Polyspecific
^182^EPPKTHVT^190^	8	0	0	19	0	0	C-restricted

HLA-E-restricted epitopes are shown in bold italics with (^*^).

HLA-E is expressed at the cell surface only if a suitable leader peptide from specific class-Ia or class-Ib alleles (other than HLA-E itself) associates as the cognate peptide of a complete HLA trimer. Some viral peptides (Table 3) can also fulfill this function. These leader peptides should be present in the ER at sufficient levels to ensure expression of a significant amount of trimeric HLA-E on the cell surface. Thus HLA-E is highly evolved to bind specifically to the class-I leader peptide.^(41)^ The class-I leader peptide or microbial (cytomegalovirus, human immunodeficiency virus, Hep-C, and others) peptides may not be available when tumor cells and virally infected cells have downregulated HLA-I expression.^(42)^ Furthermore, viruses can inhibit the function of TAP^(43,44)^, which is required to transport leader peptides, and cleaved from nascent class-I molecules, from the cytoplasm into the ER. Under these circumstances, intact HLA-E may not be expressed on the cell surface, permitting NK lysis of the tumor cell or virally infected cell.^(41)^

**Table 3.** Sources of Peptides and Their Sequences Presented by HLA-E During Interaction with CD94/NKG2

*Peptide source*	*Sequences*	*References*
HLA-B7	*VMAPTRVLL*	^(10,46,79–81)^
HLA-B27	*VTAPRTLLL*	^(46,80,81)^
HLA-Cw7	*VMAPRTLLL*	^(50)^
HLA-C^*^03:04	*VMAPRTLIL*	^(51)^
HLA-G	VMAPRTLFL	^(50)^
CMV UL40	*VMAPRTLIL*	^(82)^
	*VMAPRTLVL*	
Hepatitis C virus	*YLLPRRGPRL*	^(83)^
ABC MRP7	*ALALVRMLI*	^(84)^
HIV p24	*AISPRTLNA*	^(85)^

Sequences: A, alanine; F, phenylalanine; G, glycine; I, isoleucine; L, leucine; M, methionine; N, asparagine; P, proline; S, serine; R, arginine; T, threonine; tyrosine; V, valine.

CMV, cytomegalovirus; NKG2, natural killer cell group 2.

Two independent groups^(10,45)^ have validated the finding that HLA-E functions as the specific ligand for CD94/NKG2A receptors. The absence of binding between CD94/NKG2A and classical HLA-Ia molecules was confirmed^(46)^ by the direct binding of the soluble receptor and functional assays with CD94/NKG2A-positive NK cells. Braud et al.^(10)^ constructed *in vitro* the phycoerythrin-labeled biotinylated tetrameric complexes of the heavy chain of HLA-E and β2m with a synthetic peptide (VMAPRTVLL) derived from the signal sequence shared by HLA-B allelic proteins. The peripheral blood mononuclear cells from nine regular donors stained for the HLA-E tetramer. An HLA-A2 tetramer with an Epstein–Barr virus peptide was used as a negative control. The HLA-E tetramer bound to 2%–11% of lymphocytes from different individuals, whereas the HLA-A2 tetramer bound to 0% and 0.8% of the lymphocytes. On average, an HLA-E tetramer bound to ∼57% (varied from 35% to 83% among individuals) of CD3^−^/CD56^+^ NK cells, while the HLA-E tetramers bound to 12%–60% of CD3^+^ T cells (some were CD56^+^). Most importantly, the HLA-E tetramer did not bind to the lymphocytes when the cells were pretreated with the anti-CD94 mAb HP3D9. The interaction between HLA-E tetramers and CD94 was further confirmed by staining a number of well-characterized CD94^+^ NK-cell clones with HLA-E tetramers and demonstrating that another anti-CD94 mAb DX22 completely abolished this binding. The HLA-A2 tetramer failed to stain the CD94^+^ NK cell clones.

Lee et al.^(45)^ provided further experimental proof for the functional dynamics of the interaction between HLA-E and the NK inhibitory receptors CD94/NKG2A. The validity of their observation is based upon using two different HLA-I-deficient LCL 721.221 target cells, one expressing cytoplasmic but not cell surface HLA-E (HLA-E*0101 allele), and the other, LCL.221-AEH cells, that do express cell surface HLA-E (HLA-E*0101 allele). Besides, they used LCL 721.221 cells transfected with HLA-B*0702, B*2705, C*0401, C*0302, and B*5101 as controls. Using an effector NK cell line (CD94/NKG2A positive but negative for Ig superfamily or killer cell receptor), the effects of the presence or absence of HLA-E on the surface of LCL721.221 cells were examined. NK cell-mediated cytolysis was inhibited only when HLA-E was present on the surface of the target cells (LCL.221-AEH) but not when it is cytoplasmic (LCL 721.221). Total restoration of lysis was observed after the addition of various mAbs, including anti-HLA-E “specific” mAb 3D12 (IgG1), pan-HLA-I mAb HP-1F7 (IgG1), anti-CD94 mAb HP-3B1 (IgG2a), or anti-CD94/NKG2a mAb (Z199, IgG2b), but not with IgG1 negative control mAb. The results confirm that the inhibition of lysis was specific both for HLA-E, because protection was reversed by the 3D12 mAb, and for the CD94/NKG2A complex, because cytotoxicity was reconstituted by CD94 (HP-3B1) or CD94/NKG2A (Z199)-specific mAbs.

The results confirm the importance of HLA-E but highlight the fact that mAbs binding can abrogate the binding of NK-associated CD94/NKG2A to the extent of restoring the cytolytic capabilities of the NK cells. The observations had potential merit since a specific cell line devoid of HLA-I but incorporated explicitly with cell surface HLA-E was used. However, it is neither established whether the mAb 3D12 is capable of binding to the amino acid ligands of CD94/NKG2A nor whether mAb 3D12 is monospecific for the two well-known alleles of HLA-E (HLA-E^G107^ and HLA-E^R107^). Such in-depth analysis of monospecificity of anti-HLA-E mAbs is a prerequisite for using of anti-HLA-E mAbs for passive immunotherapy either in a preclinical model or for human trials.

We examined^(47)^ the HLA-I binding affinity of mAb 3D12 using the Luminex single antigen bead (SAB) assay with beads coated with 30 HLA-A, 50 HLA-B, 16 HLA-Cw, 2 HLA-E, 1 HLA-F, and a couple of HLA-G alleles. We found that the mAb 3D12 recognized not only HLA-E but also several HLA-Ia alleles; hence, that the mAb 3D12 is not specific for HLA-E. Therefore, a mAb that is monospecific for HLA-E and at the same time capable of binding to the amino acid ligands of CD94/NKG2A on the HLA-E molecule will be potentially useful to restore cytotoxic killing of tumor cells as well as for the specific recognition of tumor cells expressing HLA-E on the cell surface, potentially being amenable to such therapy.

## Specific Amino Acids on α1 and α2 Helices of HLA-E as Ligands for CD94/NKG2A

The disulfide-liked CD94/NKG2A dimers “sit across” the peptide-bound cleft of carboxy-terminal end of HLA-E, precisely interacting with α1 and α2 helices of HLA-E, respectively, as shown in Figure 1A. Petrie et al.^(48)^ have critically analyzed the electrostatic surfaces of HLA-E, carrying the leader sequence peptide of HLA-G namely *VMAPRTLFL* in the α1 and α2 helical groove and CD94-NKG2A. They showed that a basic region on the α1 helix of HLA-E interacted with an acidic domain on CD94 and, contrariwise, an acidic region on the HLA-E α2 helix docked with a basic domain on NKG2A. This study is restricted to the leader sequence peptide of HLA-G (residues 3–11). However, several other leader peptides sequences of HLA-Ia are known to bind to the α1 and α2 helical groove of HLA-E (Table 3). Whether the strength of acid–base interaction of NKRs and HLA-E remains the same for all these leader peptides is not clear at present, although it is assumed to be similar. However, although all HLA-I leader sequence peptides tested bound to HLA-E and were recognized by CD94/NKG2A, amino acid variations in the leader sequences affected the stability of HLA-E. It appears that CD94/NKG2A recognition of HLA-E is controlled by the degree of stabilization of peptide with HLA-E and their cell surface expression, and both HLA-E heavy chain and cognate peptide form the ligand for CD94/NKG2A. However, a comparison of the CD94/NKG2A–HLA-E *VMAPRTLFL* complex with the unligated CD94-NKG2A^(49)^ and HLA-E^(50)^ revealed no significant conformational change in either HLA-E or CD94-NKG2A upon complex formation.

According to Petrie et al.,^(48)^ the “lock and key” engagement between HLA-E *VMAPRTLFL* and CD94-NKG2A exemplified the “innate characteristic” of this interaction. The binding of CD94-NKG2A heterodimer with HLA-E involves 8 salt linkages and 19 H bonds (Table 4 and Fig. 1B, C). Besides, several van der Wall interactions are found between the heterodimer and the HLA-E (Table 4). CD94 shows greater association with α1 helix than NKG2a with the α2 helix. The CD94-NKG2a “footprints” on HLA-E was further confirmed by studying structural data, and mutating the amino acids of CD94, NKG2a, and HLA-E involved in the binding.

**Table 4.** Amino Acid Interactions Between NKG2A/CD94 and HLA-E with Leader Peptide from HLA-G

*HLA-E α1*		*HLA-E α1*		*HLA-E α2*	
*CD94*	*helix*	*CD94*	*helix*	*NKG2A*	*helix*
*H-bonds*		*van der Waal contacts*		*H-bonds*	
Ser77 = Arg65		Ser77 = Arg65		Arg137 = Ser151	
Gln112 = Glu152		Gln112 = Ile73		Lys217 = Asp162	
Gln113 = Asp69		Gln113 = Asp65			
Ser143 = Arg79		Gln113 = Asp69		*Salt linkage*	
Thr146 = Glu89		Phe114 = Glu72		Arg137 = Ser151	
Asn160 = Arg79		Phe114 = Val76		Lys199 = Asp162	
Asn160 = Arg79 (second)		Ser143 = Arg79			
Ala161 = Arg79		Thr146 = Glu89		*van der Waal contacts*	
Glu164 = Gln72		Phe147 = Arg79		Arg137 = Ser151	
Glu168 = Glu19		Asn160 = Val76		Arg137 = Glu154	
Arg171 = Arg65		Asn160 = Arg79		Lys164 = Arg65	
		Ala161 = Val76		Pro171 = His155	
*Salt linkage*		Ala161 = Arg79		Ser172 = His155	
Asp163 = Arg75		Leu162 = Gln72		Lys199 = Asp162	
Arg171 = Asp69		Leu162 = Arg75		Gln212 = Ala158	
		Leu162 = Val76		Gln212 = Glu154	
		Asp163 = Arg75		Val213 = His155	
		Glu164 = Glu19		Lys217 = Asp162	
		Glu164 = Gln72		Lys217 = Ala158	
		Asn170 = Gln72		Ser223 = Arg131	
		Arg171 = Arg65		Ser224 = Arg131	

Modified from Petrie et al.^(48)^

The interaction between the structures of the CD94-NKG2A and HLA-E*VMAPRTLIL* was compared with the αβTCR–HLA-E complex^(51)^ and that of NKG2D–MICA.^(20,23)^ The position of the CD94-NKG2A footprint on HLA-E was like that HLA-E–restricted TCR (KK50.4); however, in the KK50.4TCR interaction with HLA-E, both heavy chains of the TCR were involved equally in the binding. Similarly, the proximity of the α1 and α2 helices of MICA enabled each chain of NKG2D homodimer to interact with both helices.^(13,23)^ While comparing the HLA-E binding of the inhibitory receptor CD94-NKG2A with activating receptors CD94-NKG2C, differences in the binding affinities were observed. Both NKG2A and NKG2C show remarkable sequence differences and the binding affinity of NKG2C with HLA-E is found to be much lower than that of NKG2A.^(50,52,53)^ All these studies fundamentally clarify a critical aspect of HLA-E function, namely that there are HLA-E-specific epitopes involved in the interaction with NK or TCRs, most importantly the HLA-E-specific amino acid sequences found in the α1 and α2 helices of HLA-E. Table 4 compares the unique amino acid sequences of HLA-E-specific epitopes with other HLA class-I molecules.

## Background and the Central Hypothesis

HLA class-I molecules, which includes HLA-E, are heterodimers consisting of a heavy chain polypeptide with β2-β2m with or without a peptide in the grove. The heavy chains consist of α1, α2, and α3 helices. It is well known that HLA class-I loci include HLA-A, (with 3913 alleles and 2747 proteins), HLA-B (4765 alleles and 3465 proteins), HLA-Cw (3510 alleles and 2450 proteins), and least polymorphic HLA-E, HLA-F, and HLA-G. The genes are located on chromosome 6 (6p21.31). The heavy chain of HLA-I shares several commonly shared amino acid sequences (epitopes) and a very few specific amino acid sequences. Several shared sequences are cryptic in the heavy chain due to heterodimerization with β2m. When a trimeric HLA used as an immunogen either in animal models or when used as autologous or allogenic vaccines in patients, the shed immunogen rarely remains intact during immune recognition. Both the shed heavy chain and the β2m are recognized independently.

Consequently, upon immunizing HLA-E heavy chain in three mice, we obtained 258 mAbs. Although most of them were recognized as shared sequences and polyreactive, 31 mAbs were monospecific.^(54)^ We hypothesize that only the monospecific anti-HLA-E mAbs that are monospecific for HLA-E are not only useful for specific recognition of HLA-E on tumor cells and tissues but also can serve to prevent tumor escape from NIK cell killing by blocking by the interaction of the inhibitory NK cell CD94-NKG2A with tumor expressed HLA-E. It is critical and essential that the therapeutic anti-HLA-E mAbs generated should recognize unique HLA-E-restricted epitopes but no other epitopes are public and shared by other HLA-Ia or HLA-Ib molecules.

### Polyreactive HLA-E mAbs

**FIG. 2.**
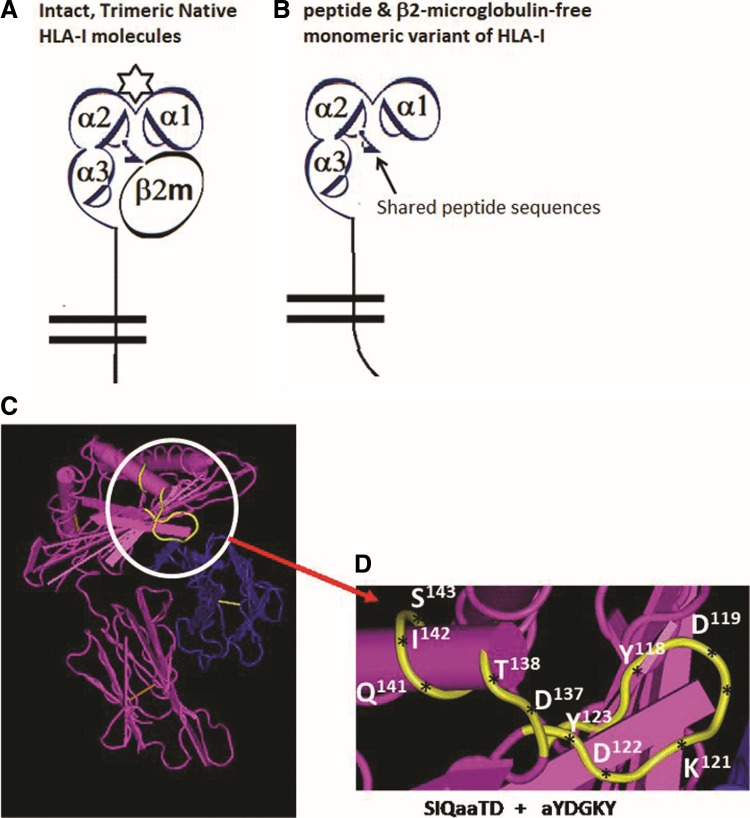
Structural variants of HLA class-I molecules. **(A)** Diagrammatic representation of an intact, native, and trimeric HLA-I molecules with α1, α2, and α3 helices of the heavy chain dimerized with β2m with the presence of a peptide (star) in the grove. The n-terminal end is extended into a bilayered lipid membrane. **(B)** Same as **(A)** but without β2m and peptide, called a monomeric variant, also known as open conformer. **(C)** Structure of an intact HLA without peptide but with β2m shown in blue. The amino acid shown in yellow is cryptic in the presence of β2m but gets exposed for immune recognition without β2m. The amino acid sequence in the yellow regions is shared by almost all HLA class-I molecules (Table 2). The monospecific mAb TFL-033 does not bind to the shared amino acid sequences. **(D)** The exact location, position, and configuration of the amino acid sequence of the shared epitopes, ^115^QFAYDGKDY^123^ and ^137^DTAAQI,^142^ recognized by the polyreactive mAb MEM-E/02 and all the anti-HLA-E mAbs included in group 8 category of mAbs are listed in Table 7. The antibody epitope prediction formulae^(62)^ support the contention that the epitope for MEM-E/02 and other MEM series is a discontinuous sequence emerging from six to seven amino acid sequences separated by a long peptide sequence of the heavy chain of HLA-E molecule.

Table 5 summarizes the literature on the commercial anti-HLA-E mAbs used for identification of HLA-E on normal and tumor tissues, and on tissues were monitored during tumor progression and metastasis. However, the commercial mAbs such as MEM-E/02, MEM-E/06, MEM-E/07, and MEM-E/08,^(55–60)^ 3D12,^(61)^ and others listed in Table 5 failed to comply with the stringent criteria of monospecificity for HLA-E, based on the binding of these mAbs to other HLA class-I antigens. This finding is well illustrated in Table 6, which shows how the most commonly used anti-HLA-E mAb MEM-E/02 and mAb 3D12 react with HLA-A, HLA-B, and HLA-Cw molecules coated on a solid matrix in the form of SABs. Each bead is coated with one of the following HLA molecules: 31 HLA-A, 50 HLA-B, and 16 HLA-Cw. Two essential and specific publications^(47,62)^ on these two mAbs clarify the notable findings in greater detail. The possible epitopes recognized by MEM-E/02 were tested at 1/1200 dilution of the mAb and by dosimetric peptide inhibition.^(47)^ Since MEM-E/02 bound to several HLA-I molecules, we have selected the epitope present in the heavy chain of HLA-E, which are most commonly shared by several HLA-I molecules, as summarized in Table 2. They are ^115^QFAYDGKDY^123^, ^137^DTAAQI,^142,^ and ^126^LNEDLRSWTA^135^. Interestingly, these shared peptides remain cryptic in intact trimeric native HLA-I (Fig. 2A), but will be exposed in β2m-free HLA heavy chain (Fig. 2B), well recognized as open conformers. Figure 2 illustrated in a prior publication^(47)^ showed that the binding of MEM-E/02 to the HLA-Ia molecules coated on the LABScreen HLA-Ia SAB beadsets^†^ is selectively inhibited by two of the shared peptides (DTAAQI [48%] and QFAYDGKDY [24%]) (Fig. 2C, D), but not inhibited by another commonly shared peptide (LNEDLRSWTA), which served as a potential control representing a shared but noninhibitory peptide. The inhibition is further confirmed by dosimetic analysis as shown in Figure 4 of the report.^(47)^ Recently, Tremante et al.^(60)^ tested the HLA-I binding of MEM-E/02 on Western blots using the purified heavy chains (NP40 lysates) from the cell lines that express HLA-A*11:01, HLA-B*35, HLA-Cw*04:01 (CJO), Cw*05:01 (221 C5), and HLA-Cw*07:01(221 C7).^(60)^ Indeed, MEM-E/02 bound to A*11:01, B*35, Cw*04:01, Cw*05:01, and Cw*07:01 as shown in our report.^(47)^ However, the authors contended that the MEM-E/02 binding to HLA-Ia antigens on Western blots is not as intense as the staining of HLA-E heavy chains. We have also reported^(47)^ that MEM-E/06, E/07 and E/08 were also cross-reactive to several other HLA class-I molecules and hence none of the MEM categories of mAbs are HLA-E monospecific.

**Table 5.** Documention Cell Surface Expression of HLA-E on Human Cancers: Monoclonal Antibodies Used Include MEM-E/02, MEM-E/06, MEM-E/07, MEM-E/08, 3D12, 3H2679, and TFL-033

*mAbs used*	*Cancer types*
MEM-E/02	Melanoma^(86)^
	Lip squamousal cell carcinoma^(87)^
	Laryngeal carcinoma^(88)^
	Vulvar intraepithelial carcinoma^(76)^
	Penile cancer^(89)^
	Glioblastomas^(77,90,91)^
	Oral osteosarcoma^(92)^
	Intraoral mucoepidermoid carcinoma^(93)^
	Rectal cancer^(94)^
	Colorectal carcinoma^(78,95–99)^
	Colon carcinoma^(97)^
	Hepatocellular carcinoma^(100)^
	Nonsmall cell lung carcinoma^(101)^
	Breast cancer^(102,103)^
	Ovarian cancer/cervical cancer^(104)^
	Cervical cancer^(105,106)^
	Cervical squamosal and adenocarcinoma^(107)^
	Serous ovarian adenocarcinoma^(108,109)^
	Renal cell carcinoma^(110,111)^
	Thyroid cancer^(112)^
	Hodgkin lymphoma^(113)^
3D12	Many cancers^(114)^
	Melanoma, cervical cancers^(115)^
	Glioblastoma stem cells^(116)^
	Glioblastoma^(117)^
	Neuroblastoma^(118)^
	Chronic lymphocytic leukemia^(119,120)^
3H2679	Neuroblastoma^(121)^
	Colorectal carcinoma^(122)^
MEM-E/06	Colon carcinoma and leukemia (K562)^(123)^
MEM-E/07 and MEM-E/08	Melanoma and other cancers^(123)^
MEM-E/08	Colorectal carcinoma^(124,125)^
TFL-033 and MEM-E/02	Gastric cancer^(126)^ melanoma^(54)^

mAbs, monoclonal antibodies.

**Table 6.** Anti-HLA-E mAbs Reported To Be “Specific” for HLA-E (MEM-E/02 and 3D12) Cross-React with Several HLA-Ia Molecules

*HLA alleles*	*MEM-E/02*	*3D12*	*HLA alleles*	*MEM-E/02*	*3D12*	*HLA alleles*	*MEM-E/02*	*3D12*
A^*^1101 (A11)	5171	2372	B^*^1510 (B71)	1566		B^*^5101 (B51)	3518	
A^*^2402 (A24)	2072		B^*^1511 (B75)	4917		B^*^5102 (B51)	1915	
A^*^2403 (A24)	1034		B^*^1513 (B77)	3738		B^*^5201 (B52)	4137	
A^*^2601 (A26)	2873		B^*^1516 (B63)	1451		B^*^5301 (B53)	6999	2960
A^*^2901 (A29)	1319		B^*^1801 (B18)	2996		B^*^5401 (B54)	3784	
A^*^3001 (A30)	1013		B^*^2708 (B27)	1012		B^*^5501 (B55)	3980	
A^*^3002 (A30)	1272		B^*^3501 (B35)	2219		B^*^5601 (B56)	2014	
A^*^3203 (A32)	1204		B^*^3701 (B37)	7514		B^*^5701 (B57)	1724	
A^*^3301 (A33)	1575		B^*^3801 (B38)	1325		B^*^5703 (B57)	2193	1032
A^*^3301 (A33)	1561		B^*^4001 (B60)	4912	1328	B^*^5801 (B58)	5683	3126
A^*^3401 (A34)	1950		B^*^4002 (B61)	2771		B^*^5901 (B59)	5112	1303
A^*^4301 (A43)	1010		B^*^4006 (B61)	9540	4899	B^*^7301 (B73)	2255	
A^*^8001 (A80)	1161		B^*^4101 (B41)	4040		B^*^7801 (B78)	2947	
B^*^0801 (B08)	1858		B^*^4402 (B44)	5330		B^*^8101 (B81)	1898	
B^*^1301 (B13)	5811	1720	B^*^4403 (B44)	4010	1011	B^*^8201 (B82)	5700	
B^*^1302 (B13)	4699	1118	B^*^4501 (B45)	1691				
B^*^1401 (B64)	5653	1677	B^*^4601 (B46)	2652				
B^*^1402 (B65)	2624		B^*^4701 (B47)	2684		Both mAbs failed to react		
B^*^1502 (B75)	2675		B^*^4801 (B48)	6294		With iBeads containing only		
B^*^1503 (B72)	5619		B^*^4901 (B49)	1388		With β2aHC (MFI <1000).		

The mAbs were diluted 1/100. Only those alleles with MFI above >1000 are presented. The mAbs were tested on Luminex SABs assay using regular LABSCreen beadsets (Lot-7) and with iBeads. The regular beadsets are coated with both β2m-associated, peptide–associated, or peptide-free heavy chain (β2aHC) with admixture of β2m-free heavy chains of HLA (β2fHC).^(63)^

β2m, β2-microglobulin; MFI, mean fluorescent intensity; SABs, single antigen beads.

Most interestingly, none of these MEM series antibodies bound to HLA-I antigens coated on a modified version of the regular beads called iBeads. The regular beadsets provided for the Luminex assay by the manufacturer (One Lambda, Inc., Canoga Park, CA) are coated not only with intact trimeric HLA class-I molecules but also contain free α-heavy chains of HLA class-I without β2m and/or the cognate peptide^(63)^ (Fig. 2B). Realizing the existence of the monomeric and dimeric HLA-I variants in addition to intact trimeric HLA molecules on the beadsets, the manufacturer developed iBeads, provided as felix beads for in-house (at Terasaki Foundation Laboratory [TFL]) experimental use. Although the iBeads have been well investigated in clinical laboratories^(63–70)^ and proven to be specific for containing only intact trimeric HLA-I molecules (Fig. 2A), their commercial production was abandoned as it was not cost-effective for commercial use. The iBeads carried only the intact trimeric HLA. These iBeads are produced by proprietary enzymatic treatment of regular HLA-Ia antigen coated microbeads to remove or reduce the amount of free heavy chains (also referred to as “denatured antigens”) by the manufacturers. Most interestingly, the mAb MEM-E/02 failed to bind to any one of the HLA molecules coated on the 97 different iBeads carrying HLA class-I alleles. This finding suggests that MEM-E/02 is not capable of reacting to intact cell surface HLA-E or HLA class-I molecules (Fig. 2A), but binds only to the heavy chain open conformers lacking association with β2m and/or peptide (Fig. 2B). We have further examined this issue with another mAb3D12,^(62)^ considered to be specific for HLA-E,^(61)^ by measuring the mean fluorescence intensity (MFI) with regular SABs. The results showed that 3D12 simulated MEM-E/02 in recognizing several HLA-B and HLA-C antigens. As observed with MEM-E/02, binding of 3D12 to HLA-E is inhibited by the public peptide sequences QFAYDGKDY and DTAAQI, shared by other class-Ia and class-Ib antigens. Furthermore, a decrease in binding of mAb 3D12 to HLA class-Ia SABs after heat treatment supports the contention that the epitope is located at the outside of the “thermodynamically stable” α-helix conformations of HLA-E. Therefore, we emphasize that reliable immunodiagnosis of HLA-E on histopathological studies on tissues, mainly derived from cancer patients, require proof of monospecificity of the mAb for HLA-E, by the dosimetric inhibition of the HLA-E mAbs with one or more of the HLA-E monospecific epitopes listed in Tables 1 and 2.

### Monospecific HLA-E mAbs

**FIG. 3.**
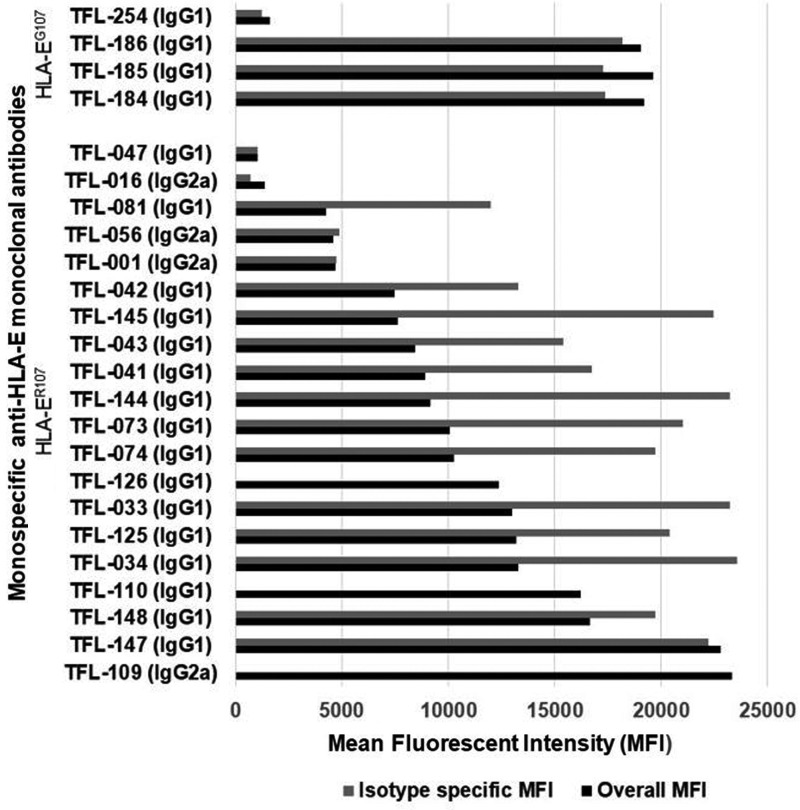
Several hybridoma clones secreting monospecific mAbs were generated by immunizing the heavy chains of either HLA-E^R107^ or HLA-E^G107^. The number of clones secreting the mAbs in general and the monospecific mAbs in particular were much higher with HLA-E^R107^ than with HLA-E^G107^. The most common isotype seems to be IgG1. The MFI of the culture supernatants as well the MFI of IgG isotypes is indicated in the figure. For detailed investigation, we have used TFL-033. Ig, immunoglobulin; MFI, mean fluorescent intensity.

**FIG. 4.**
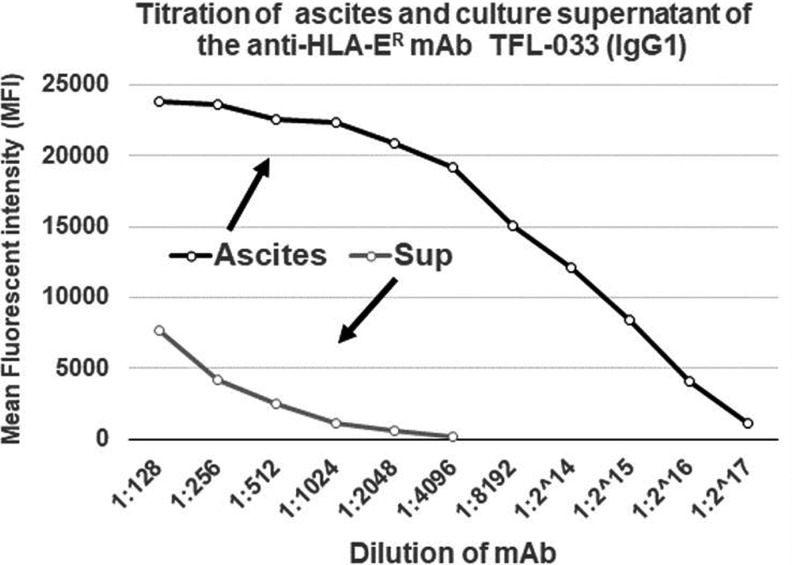
Titration of ascites and culture supernatant of the anti-HLA-ER mAb TFL-033 (IgG1). ER, endoplasmic reticulum.

Since the commercial anti-HLA-E mAbs, particularly those most commonly used on human cancer tissues such as MEM-E/02 and 3D12 (Table 5), do not meet the critical requirement of monospecificity of the mAb, they may not be considered reliable for specific immunorecognition of HLA-E on human cancer tissues. An imminent need is recognized to generate anti-HLA-E mAbs that can bind only to amino acid sequences (epitopes) unique or specific for HLA-E and to revalidate the expression of HLA-E on the cell surface of cancer cells. Therefore, we have generated^(54)^ several hybridoma clones (*n* = 258) secreting anti-HLA-E mAbs using recombinant heavy chains of the two different alleles of HLA-E, namely HLA-E^G107^ and HLA-E^R107^. The glycine (G) at position 107 is also seen with HLA-F, HLA-G, and several other HLA-Ia alleles,^(54)^ whereas arginine 107 is extremely rare among HLA-I, with two exceptions, HLA-A*01:01:01:02N and HLA-B*15:30. These mAbs were investigated for their specific affinity for HLA-E by measuring their MFI in Luminex SAB assays using beads coated with 31 HLA-A, 50 HLA-B, 16 HLA-Cs, one HLA-G, and one HLA-F antigen. Based on the analysis of the affinity of the antibodies to HLA-Ia and HLA-Ib molecules, the mAbs were categorized into eight groups as defined in Table 7. Group 1 of the eight groups constitutes mAbs that do not react with HLA class-Ia alleles or with HLA-F and HLA-G. However, they reacted to both HLA-EG and HLA-ER. The MFI of the isotypes of group 1 is compared to assess the potential strength of the mAbs (Fig. 3). The number of clones secreting the mAbs (MFI ≥1000) in general and the monospecific mAbs (MFI >1000) were much higher with HLA-E^R107^ (12 per mouse) than with HLA-E^G107^ (8 per mouse). The most common isotype is IgG1. Several clones showed remarkable proliferation in culture. The culture supernatants of five of these clones (TFL-033, TFL-034, TFL-073, TFL-074, and TFL-145) were purified using Protein-G columns tested for antibody titers (Table 8).^(54)^ TFL-033 was selected for analysis of both ascites and culture supernatants. The titrimetric profile of TFL-033 is shown in Figure 4.

**Table 7.** HLA-E Heavy Chain Monomer, Upon Immunization in a Mouse Model, Elicits Eight Different Groups of Anti-HLA-E Antibodies; However, Only Group 1 Befits the Definition of HLA-E-Specific Monoclonal Antibody Because of Its Monospecificity

*Classification of the anti-HLA-E mAbs generated at TFL using heavy chain of HLA-E*							
*Anti-HLA mAbs*	*Classical HLA (HLA-Ia)*			*Nonclassical HLA (HLA-Ib)*			*Characteristics of the groups of mAbs*
*Alleles ->*	*A*	*B*	*Cw*	*E*	*F*	*G*	
Group 1	0	0	0	+	0	0	HLA-E monospecific
Group 2	0	0	0	+	+	0	HLA-F cross-reactive
Group 3	0	0	0	+	0	+	HLA-G cross-reactive
Group 4	0	0	0	+	+	+	HLA-Ib specific
Group 5	+	+	+	+	0	0	HLA-Ia polyreactive
Group 6	+	+	+	+	+	0	HLA-Ia/F polyreactive
Group 7	+	+	+	+	0	+	HLA-Ia/G polyreactive
Group 8	+	+	+	+	+	+	HLA class-I specific

The monospecific monoclonal HLA-E antibodies recognize none of the other HLA-Ia or Ib molecules. TFL refers to Terasaki Foundation Laboratory in Los Angeles, where the antibodies were generated and characterized. The mAbs were monitored in Luminex SABs assay using regular LABSCreen beadsets.

**Table 8.** Titers of Protein-G Purified Culture Supernatants of Monospecific Anti-HLA-E^R^

*mAbs*	*Protein-G second eluate (protein concentrationn g at 1/10 dilution)*	*Titer*
TFL-033	44	1000
TFL-034	45	500
TFL-073	40	200
TFL-074	56	300
TFL-145	46	12,000

Titer of the mAbs measured after serial dilution of the immunogen used is the heavy chain of HLA-E.

### Immunodiagnostic potential of a monospecific anti-HLA-E mAb

**FIG. 5.**
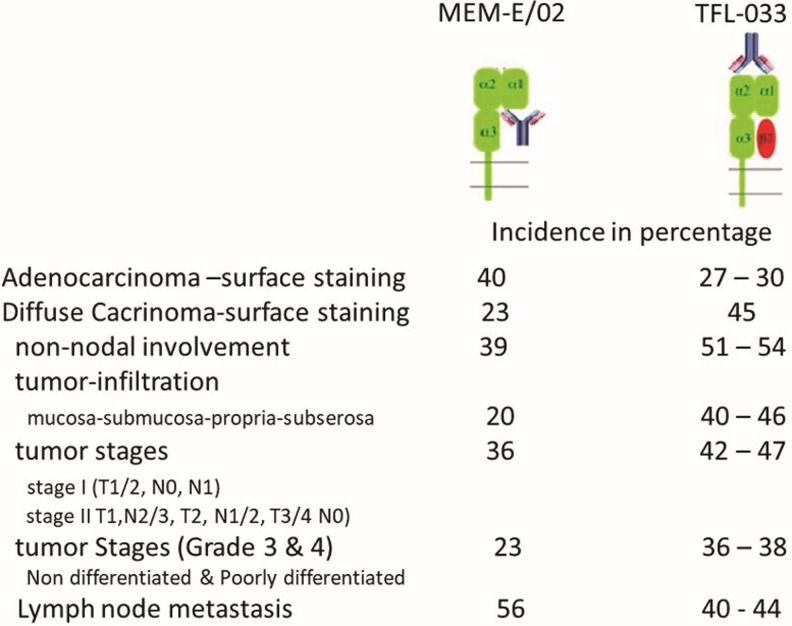
Comparative staining of gastric adenocarcinoma and diffuse carcinoma with trimeric HLA-E reactive monospecific mAb TFL-033 and β2m-free heavy chain of HLA-E and HLA-I polyreactive mAb MEM-E/02. Please note that intense staining or higher incidence of staining with MEM-E/02 does not imply it is due to intact, trimeric, or dimeric HLA-E but due to the monomeric heavy chain of HLA-I including HLA-E. In adenocarcinoma, MEM-E/02 stained 12 of 30 tissue samples (40%) and the incidence is higher than that of TFL-033 (27%–30%). In diffuse carcinoma, TFL-033 stained 18 of 40 tissue samples (45%). The incidence is higher than that of MEM-E/02 (23%). The node-negative was significantly more positive staining with TFL-033 than with the node-positive. Metastatic cancer included peritoneal (*n* = 5), liver (*n* = 3), ovarian (*n* = 5), and lymph node (*n* = 27) metastases. With the mAb MEM-E/02, 21 of the 40 metastatic carcinomas stained intensely (53%), higher than that of TFL-033 (38%–40%). With lymph node metastasis, the incidence of staining by TFL-033 was 40%, whereas that of MEM-E/02 is 56.6%. [Source: Sasaki et al.^(71)^].

The purified fraction of the TFL mAbs was used for comparative staining with MEM-E/02 on tissues of human gastric cancer^(71)^ and melanoma.^(54)^ As noted earlier, HLA-E without a leader peptide in the grove does not give stability to the α-heavy chain/β2m heterodimer. However, as noted in our report,^(54)^ of the eight groups of mAbs, only one group is truly specific for HLA-E, whereas others bind to epitopes shared with other HLA loci, such as HLA-A, HLA-B, HLA-Cw, HLA-F, and HLA-G. The mAb TFL-033-specific epitopes occur in α1 helix (which form the groove) and at adjacent naturally exposed domain on the helices, as illustrated in Table 9 and Figure 1A and B. The mAb TFL-033 binding to HLA-E coated on SABs was tested using such HLA-E monospecific peptides in a dosimetric manner, and the results are illustrated in detail in a figure [fig. 5 in Ravindranath et al.^(54)^]. The dosimetric inhibition of purified culture supernatants of TFL-033 was examined with two HLA-E-restricted peptides, ^65^RSARDTA^71^ and ^143^SEQKSNDASE^152^, at concentrations ranging from 4.4 to 0.27 mg/well. Although both peptides showed inhibition, the α2 helical peptide SEQKSNDASE showed higher inhibition than the other peptide. These findings suggest that TFL-033 has better specificity for recognition of intact trimeric HLA-E than most of the polyreactive commercial mAbs, which bind to β2m-free HLA-E. That is one of the reasons why MEM-E/02 did not bind to iBeads (the regular LABSCreen beadset enzymatically treated to eliminate monomeric and dimeric variants of HLA-I) but bound only to the regular LABScreen beads. Since human cancer cells can express both intact trimeric HLA and β2m-free HLA, there is a need to distinguish the different phenotypic expression of HLA-E on the surface of tumor lesions. Because of the unique peptide-binding affinities of TFL-033 and MEM-E/02, we used both mAbs to study this aspect in gastric cancer^(71)^ and melanoma.^(54)^ Only TFL-033 stained the cytoplasm of normal mucosa (Fig. 5) diffusely. The incidence and intensity of staining of the cell surface in early stages, poorly or undifferentiated and non-nodal lesions by TFL-033 are markedly higher than the staining by MEM-E/02. Of note, however, MEM-E/02 stained terminal stages of adenocarcinoma and lymph node metastatic lesions intensely, either owing to increased expression of β2m-free HLA-E with tumor progression or owing to expression of other β2m-free HLA-Ia molecules. Similar comparative observations made on human melanoma tissues^(54)^ validate the hypothesis that monospecific anti-HLA-E mAbs reveal the presence of intact trimeric native HLA-E than the commercial polyreactive nonspecific HLA-E mAbs.

**Table 9.** Comparison of the HLA-E Monospecific Amino Acid Sequences (Epitopes) of Three Epitopes (One 6-mer [#1], One 9-mer [#2], and One 5-mer [#3])

*HLA-E restricted peptides (#1) and (#3)*											
*HLA-Ib alleles*	*Amino acid positions at α1 heavy chain of HLA*										
	*Peptide (#1)*						*Peptide (#3)*				
	65	66	67	68	69	70	180	181	182	183	184
E^*^01010101	**R**	**S**	**A**	**R**	**D**	**T**	**L**	**H**	**L**	**E**	**P**
G^*^01010101	**R**	N	T	K	A	H	Q	R	A	D	**P**
F^*^01010101	G	Y	**A**	K	A	N	Q	R	A	D	**P**
A^*^110101	R	N	V	K	A	O	Q	R	T	D	**P**
B^*^1401	Q	I	C	K	T	N	Q	R	A	D	**P**
B^*^350101	Q	I	F	K	T	N	Q	R	A	D	**P**
B^*^40060101	Q	I	S	K	T	N	Q	R	A	D	**P**
B^*^530101	Q	I	F	K	T	N	Q	R	A	D	**P**
B^*^5801	R	N	M	K	A	S	Q	R	A	D	**P**
Cw^*^050101	Q	K	Y	K	R	Q	Q	R	A	**E**	H
Cw^*^080101	Q	K	Y	K	R	Q	Q	R	A	**E**	H
Cw^*^1802	Q	K	Y	K	R	Q	Q	R	A	**E**	H
Qa-1	W	K	A	R	**D**	M	Q	R	S	**E**	**P**

*HLA-Ib alleles*	*Amino acid positions at α1 heavy chain of HLA*								
	*Peptide (#2)*								
	*143*	*144*	*145*	*146*	*147*	*148*	*149*	*150*	*151*
E^*^01010101	**S**	**E**	**Q**	**K**	**S**	**N**	**D**	**A**	**S**
G^*^01010101	**S**	K	R	**K**	C	E	A	**A**	N
F^*^01010101	T	Q	R	F	Y	E	A	E	E
A^*^110101	T	K	R	**K**	W	E	A	**A**	H
B^*^1401	T	Q	R	**K**	W	E	A	**A**	R
B^*^350101	T	Q	R	**K**	W	E	A	**A**	R
B^*^40060101	T	Q	R	**K**	W	E	A	**A**	R
B^*^530101	T	Q	R	**K**	W	E	A	**A**	R
B^*^5801	T	Q	R	**K**	W	E	A	**A**	R
Cw^*^050101	T	Q	R	**K**	W	E	A	**A**	R
Cw^*^080101	T	Q	R	**K**	W	E	A	**A**	R
Cw^*^1802	T	Q	R	**K**	W	E	A	**A**	R
Qa-1	**S**	K	R	**K**	S	E	A	V	D

*HLA-E restricted peptide (#1)*						
*HLA-Ib alleles*	*180*	*181*	*182*	*183*	*184*				
E^*^01010101	**R**	**S**	**A**	**R**	**D**				
G^*^01010101	**R**	N	T	K	A				
F^*^01010101	G	Y	**A**	K	A				
A^*^110101	R	N	V	K	A				
B^*^1401	Q	I	C	K	T				
B^*^350101	Q	I	F	K	T				
B^*^40060101	Q	I	S	K	T				
B^*^530101	Q	I	F	K	T				
B^*^5801	R	N	M	K	A				
Cw^*^050101	Q	K	Y	K	R				
Cw^*^080101	Q	K	Y	K	R				
Cw^*^1802	Q	K	Y	K	R				
Qa-1	W	K	A	R	**D**				

Since each locus has >1000 alleles, due to space constraints, few alleles for each locus were selected. Qa-I, a murine equivalent of hu HLA-E is also compared. Peptide #1 and peptide #2 were selected for diametric inhibition of one of the monospecific mAbs [TFL-033, see fig. 5 in Ravindranath et al.^(54)^]. The term monospecificity implies the binding of the mAbs to a unique epitope of HLA-E (Table 4).

The bold letters of amino acids of alleles other than HLA-E^*^01010101 indicate the corresponding position of the amino acids found in HLA-E. The lack of repetition of the entire sequence of amino acids found in HLA-E establish that the amino acid sequence is HLA-E restricted.

### Potential of monospecific anti-HLA-E mAb to avert CD94/NKG2A interaction

The binding domain of the monospecific anti-HLA-E mAb (e.g., TFL-033) and CD94 and NKG2A on the α1 and α2 helices of HLA-E is the same. Both TFL-003 and the CD94/NKG2A heterodimer share some of the specific amino acids. However, we have not done peptide inhibition studies on all the monospecific anti-HLA-E mAbs. It is reasonable to infer that all of them may bind to HLA-E-specific epitopes, which could exist on the α1 and a2 helices of HLA = E. Possibly some mAbs may share more amino acids in the binding domain with the NK-inhibitory receptors, CD94-NKG2A, that react with HLA-E-specific epitopes on the α1 and α2 helices. Figure 1A illustrates the exact amino acid (in box) and its location in the epitope in the α1 helix, with either TFL-033 or CD94. Figure 1B documents the exact amino acid (in box) and its location in the epitope in the α2 helix that reacts with both TFL-033 and NKG2A. The nature of the interaction of CD94 or NKG2A with their respective binding domains on the helices is further clarified in Figure 1A and B as well as in Table 4. Two critical issues should be addressed in this context. CD94 or NKG2A bind to more than one amino acid in their binding domain, but TFL-033 binds to only one of those amino acids recognized by CD94 or NKG2A. Can such binding to a single amino acid in each helix by TFL-033 suffice to block the interaction of CD94 or NKG2A? It should be noted that for peptide inhibition studies on TFL-033, we have employed only the amino acids specific for HLA-E found on each helix. We did not test the whole amino acid sequence recognized by CD94 or NKG2A. Probably it would have provided better inhibition of TFL-033 binding to HLA-E on beads. Second, if a mAb binds to that epitope, it is sure to create stearic hindrance to block the binding of CD94 and NKG2A. Possibly other monospecific mAbs (*n* = 24) may differ in their efficacy to block binding of CD04/NKG2A binding based on the amino acid sequences recognized by the mAbs. Examining the nature of amino acids that are recognized by TFL-033, it may be noted in Figure 1A that Arginine [R] at position-65 constitutes the part of the epitope recognized by mAb TFL-033. The interaction of CD94 with R-65 involves to hydrogen bonding and three van der Waal forces (see Table 4 for details). Monospecific mAb binding can cause hindrance for this powerful interaction between R65 and CD94.

Similarly, serine [S] at position 152 recognized by the mAb involves both H bonding and van der Waal forces of interaction between NKG2A and S-152. However, it may be that the molecular size and configuration of the IgG1 mAbs that have spread and bound on the helices of HLA-E are sufficient to prevent the interaction between HLA-E and the inhibitory receptors. On blocking the binding of the inhibitory receptors on NK/CD8^+^ T cells, the antitumor cytotoxic functions of these immune cells are restored.

Another important issue is whether TFL-033 is capable of binding to the epitopes on the two helices when a peptide is present in the grove. The fact that CD94 and NKG2A involve in a variety of H bonding with salt linkage and multiple van der Waal force bindings with a single amino acid (see Table 4 for details) even in the presence of a peptide in the grove suggests that TFL-033 would also bind. As indicated in Figure 1A and B, the mAb binding sites are exposed at the periphery of grove peptide binding sites.

Therefore, a humanized or human monospecific anti-HLA-E mAb mimicking TFL-033 is bound to be a valuable agent for passive immunotherapy of human cancers. It is well known that the therapy benefits not all of the patients receiving the current NK immunotherapy protocols. Failure of patients to respond to NK therapy could be due to the level of expression of HLA-E on the tumor cell surface. If highly expressed, NK cells may be ineffective. The report provides hope that a combinational therapy admixed with anti-HLA-E monospecific mAb can be beneficial for these patients.

## Monospecific Anti-HLA-E mAbs Promotes Proliferation of CD8^+^ T Cells

The monospecific anti-HLA-E mAbs had the potential to augment the proliferation of nonactivated and activated CD3^+^/CD8^+^ cytotoxic T cells and CD3^−^/CD8^+^ NK cells or NKT cells.^(54)^ Almost all monospecific mAbs augmented the proliferation of phytohemagglutinin (PHA)-untreated T lymphoblasts (TFL-033s at 1/30 (*p* < 0.02) and 1/150 (*p* = 0.001), TFL-034 at 1/10 (*p* = 0.005) and 1/50 (*p* = 0.002), TFL-073 at 1/50 (*p* = 0.001), TFL-074 at 1/10 (*p* = 0.006) (Table 10). The specific increase of “PHA-untreated” T lymphoblasts points out the potential of the monospecific anti-HLA-E mAbs in augmenting CD8^+^ T lymphoblasts. In addition, a significant increase in the blastogenesis of CD8^+^ T lymphoblasts was observed among the PHA-treated T lymphoblasts with TFL-033 at 1/30 (*p* < 0.02) and 1/150 (*p* = 0.003), with TFL-034 at 1/50 (*p* < 0.01), and with TFL-145 at 1/100 (*p* = 0.05). None of the other CD3^+^ T cells (CD4^+^/CD8^−^, CD4^−^/CD8^−^, and CD4^+^/Cd8^+^) PHA untreated or PHA treated showed any change in the number of lymphoblasts. We have also used a polyreactive anti-HLA-E mAb TFL-007 as control, decreasing the population of untreated CD3^+^ and CD8^+^ T lymphoblasts (Table 10). This unique property of augmenting the number of CD8^+^ T lymphocytes and not other lymphocytes (Table 10) by monospecific but not by polyreactive anti-HLA-E mAbs strongly clarifies the potential of the monospecific anti-HLA-E mAbs for passive immunotherapy of cancer. Immunodiagnostic observations and peptide inhibition studies reported earlier point out that monospecific anti-HLA-E TFL mAbs are indeed capable of binding to HLA-E molecules expressed on the CD8^+^ T and NK cells. HLA-E is a component of unspecified HLA class-I antigens earlier identified at low levels on inactivated CD8^+^ cells and upregulated^(72,73)^ or heavily clustered^(74)^ in activated (by PHA or IFNγ) CD8^+^ cells. Both blastic transformation and proliferation of immune cells induces transient expression of cell-surface molecules. These molecules include IL-2R, Fc receptors for IgG (FcγRI/CD64, FcγRII/ CD32 and FcγRIII/CD16, IgE (FcɛRII)/CD23), insulin receptors; insulin-like growth factor 1R and IL-2R, alpha-fetoprotein and transferrin receptors, a non-disulphide-linked heterodimer of polypeptide chains 33 kDa and 38 kDa called ‘Me14/D12’, MICA, and HLA class II antigens HLA-DR, -DP and -DQ [for detailed information and references^(75)^]. Most importantly, over-expression of β2m-free heavy chains of HLA class I are documented^(76–78)^). It is anticipated that the binding of the monospecific anti-HLA-E mAb onto α1 and α2 helices of the overexpressed and clustered open conformers of HLA-E^(74)^ in activated normal human CD8^+^ T and NKT cells may induce phosphorylation, promoting proliferation of both nonactivated and PHA-activated CD8^+^ T and NKT cells. A model illustrating the hypothesis is presented in our previous report.^(54)^ Since NKT cells are devoid of CD3 molecules, it is envisaged that the TFL mAb-mediated activation of CD8^+^ T cells and NKT cells may be independent of CD3 molecules or may involve different cell surface receptors.

**Table 10.** Experimental Analysis of Proliferation of CD3^+^/CD8^+^ T Cells with Anti-HLA-E Monospecific mAbs (TFL-033, TFL-034, TFL-073, TFL-074, TFL-145) and an Anti-HLA-E Polyreactive mAb (TFL-007) as the Control

*Dilutions*	*No mAb (*n* = 5)*														
	*CD3^+^ native cells*					*CD3^+^ lymphoblasts*									
		*No PHA*		*With PHA*		*No PHA*				*Total*	*With PHA*				*Total*
		*CD4^+^/CD8^−^*	*CD4^+^/CD8^−^*	*CD4^+^/CD8^−^*	*CD4^−^/CD8^+^*	*CD4^+^/CD8^−^*	*CD4^−^/CD8^+^*	*CD4^+^/CD8^+^*	*CD4^−^/CD8^−^*		*CD4^+^/CD8^−^*	*CD4^−^/CD8^+^*	*CD4^+^/CD8^+^*	*CD4^−^/CD8^−^*	
	M	3063	547	1249	475	197	65	141	52	454	867	325	128	289	1609
	SD	149	86	99	37	33	14	35	15	70	115	126	43	84	267
	*p*^2^	NS	NS	<0.0001	NS	NS	NS	NS	NS	NS	0.001	0.001	NS	0.001	0.001
*TFL-033 (IgG1) (*n* = 3) monospecific peptide (epitope) inhibition tested (tested dilution 1/3)*															
1/30	M	3185	755	1170	536	223	**163**	153	99	414	1129	**505**	152	412	2197
	SD	180	146	58	12	40	27	80	13	120	86	23	16	20	139
	*p*^2^	NS	NS	NS	0.009	NS	**0.015**	NS	0.005	NS	0.01	**0.016**	NS	0.014	0.004
1/150	M	3238	681	1149	508	252	**120**	205	68	645	1266	**572**	157	412	2407
	SD	14	64	21	22	30	17	13	9	28	80	31	14	16	116
	*p*^2^	NS	NS	NS	NS	0.05	**0.001**	0.02	NS	0.003	0.001	**0.003**	NS	0.001	0.001
*TFL-034 (IgG1) (*n* = 3) monospecific peptide inhibition not tested*															
1/10	M	3048	631	1140	437	213	**114**	182	71	580	801	322	117	207	1446
	SD	83	90	158	54	16	24	10	22	44	97	29	2	36	162
	*p*^2^	NS	NS	NS	NS	NS	**0.005**	0.003	NS	0.015	NS	NS	NS	NS	NS
1/50	M	3354	687	1098	445	229	**121**	186	104	640	1424	**602**	160	362	2547
	SD	132	26	26	17	35	20	14	14	45	142	39	9	24	197
	*p*^2^	0.03	0.009	0.012	NS	NS	**0.002**	0.02	NS	NS	0.007	**0.009**	NS	NS	0.001
*TFL-073 (IgG1) (*n* = 3) monospecific peptide inhibition not tested*															
1/10	M	2850	440	1089	374	153	63	137	58	411	682	307	107	209	1285
	SD	82	14	132	36	23	1	15	19	14	78	33	8	40	152
	*p*^2^	NS	NS	NS	0.03	NS	NS	NS	NS	NS	NS	NS	NS	NS	NS
1/50	M	3119	637	1053	437	239	**120**	208	82	543	1009	**472**	140	425	2046
	SD	72	53	80	49	25	**14**	24	15	47	105	**60**	16	119	287
	*p*^2^	NS	NS	0.02	NS	NS	**0.001**	0.02	0.03	0.004	NS	NS	NS	NS	NS
*TFL-074 (IgG1) (*n* = 3) monospecific peptide inhibition not tested*															
1/10	M	2933	362	1252	515	214	**106**	201	66	587	840	363	141	278	1623
	SD	95	304	75	51	13	**18**	18	9	25	119	3	20	56	179
	*p*^2^	NS	NS	NS	NS	NS	**0.006**	0.03	NS	0.017	NS	NS	NS	NS	NS
1/50	M	3401	624	1193	521	178	72	130	55	435	751	361	74	257	1444
	SD	28	13	8	17	27	13	15	3	40	54	64	19	52	273
	*p*^2^	0.007	NS	NS	NS	NS	NS	NS	NS	NS	NS	NS	NS	NS	NS
*TFL-145 (IgG1) (*n* = 3) monospecific peptide inhibition not tested*															
1/20	M	3349	728	1206	500	173	83	157	47	459	771	**349**	97	257	1474
	SD	169	50	58	85	18	6	27	2	43	61	**14**	6	43	36
	*p*^2^	NS	0.006	NS	NS	NS	NS	NS	NS	NS	NS	NS	NS	NS	NS
1/200	M	3200	527	1160	410	238	76	168	66	548	1118	**480**	118	282	1998
	SD	229	37	14	30	32	6	9	9	12	65	**71**	23	74	271
	*p*^2^	NS	NS	NS	0.038	NS	NS	NS	NS	NS	NS	**0.05**	NS	NS	NS
*TFL-007 (IgG1) (*n* = 3) HLA-I polyreactive nonspecific anti-HLA-E mAb as control*															
1/10	M	2876	451	1183	444	164	**63**	145	52	424	676	**317**	100	222	1315
	SD	135	72	19	26	33	**2**	3	17	47	79	**25**	4	29	125
	*p*^2^	NS	NS	NS	NS	NS	NS	NS	NS	0.027	NS	NS	NS	NS	NS
1/50	M	3088	667	1075	491	230	**107**	193	80	610	892	**443**	122	339	1795
	SD	65	16	55	49	23	**7**	17	4	26	26	18	**8**	21	38
	*p*^2^	NS	0.018	0.013	NS	NS	**0.0005**	0.019	0.006	0.002	NS	NS	NS	NS	NS

Peripheral blood lymphocytes were obtained from a normal healthy volunteer. Both PHA-untreated and PHA-treated CD4^*−*^/CD8^+^ cells but not CD4^+^/CD8^*−*^ or CD4^+^/CD8^+^ or CD4^*−*^/CD8^*−*^ cells are induced proliferation only by anti-HLA-E monospecific mAbs. The experimental protocols are described in detail elsewhere.^(125)^ Paired test was performed between No mAb values versus TFL-mAb corresponding values. Significant values in the column of CD4^*−*^/CD8^+^ are shown in bold.

Ig, immunoglobulin; mAb, monoclonal antibody; NS, nonsignificant.

## Conclusions

HLA-E is one of the MHC class-I antigens with structural configuration identical to most of the other classical HLA-Ia and nonclassical HLA-Ib molecules. Using polyreactive anti-HLA-E mAbs, enumerable studies suggest overexpression of HLE-E on the cell surface of several human cancers. We have confirmed the overexpression of HLA-E on the cell surface of gastric cancers and melanoma, using monospecific anti-HLA-E mAbs.^(54,71)^ Tumor-associated HLA-E, upon binding to the inhibitory receptors (CD94/NKG2A) on NK cells or cytotoxic T cells (CD8^+^), inactivates the ability of such cells to kill tumor cells. Possibly for this reason, the efficacy or outcome of NK and CD8^+^ T cell-mediated cancer treatment is not always as anticipated. We have developed two categories of mouse HLA-E mAbs, monospecific and polyreactive HLA-E mAbs.^(54)^ Monospecific HLA-E mAbs bind to HLA-E only, but not to any other HLA-Ia or HLA-Ib molecules. In contrast, polyreactive HLA-E mAbs cross react with three or more of the other loci of HLA class-I, namely HLA-A, HLA-B, HLA-C, HLA-F, and HLA-G, which is similar to commercially available mAbs (such as MEM-E0/2, MEM- E0/6, MEM-E0/7, MEM-E0/8 and 3D12), Therefore, the monospecific anti-HLA-E mAbs are better able to confirm specific HLA-E expression on tumor cells and biopsies obtained from cancer patients. Furthermore, peptides from the amino acid sequences located on the α1 and α2 helices of HLA-E specifically inhibited the binding of the monospecific mAbs. Interestingly, the same sequences are also associated with the interaction of HLA-E with the inhibitory receptors (NKG2A and CD94) of NK and CD8^+^ cells. Since both the monospecific mAbs such as TFL-033 and inhibitory receptors NKG2A/CD94 on NK and cytotoxic T cells bind to the same sequence of the peptides located in the HLA-E α1 and α2 helices, these HLA-E-specific mAbs may have the potential to block the inhibitory pathway responsible for the poor outcome of the NK and CD8^+^ T cell-based cytotoxic killing of cancer cells in some cases. Furthermore, these anti-HLA-E monospecific but not polyreactive mAbs (such as TFL-007) have the potential to stimulate the blastogenesis and proliferation of the cytotoxic CD8^+^ T cells.^(54)^ Therefore, the anti-HLA-E monospecific HLA-E mAbs have dual antitumor potential, both to release NK and CD8^+^ T cells from their inactive state caused by binding of CD94.NKG2A with HLA-E and to simultaneously augment proliferation of the cytotoxic CD3^+^/CD4^−^/CD8^+^ T lymphocytes.

Thus, the monospecific anti-HLA-E mAbs can enhance the immune cell-based therapies paving the way for a better clinical benefit. In addition, these monospecific anti-HLA-E mAbs may also represent a novel prognostic tool in patients with GI tumors or malignant melanoma and other metastasized cancers who plan to undergo immune cell (NK/NKT/CD8^+^ T cell)-based therapies by identifying which tumors/patients are most likely to respond. Preclinical and clinical trials are required to define the proper role of this technology in the prognostication and treatment of these common cancers. In principle, we suggest that anticancer NK immunotherapy requires determination of the degree of expression of HLA-E on patients' primary or metastatic tumors, with higher expression indicating those most likely to respond to monospecific anti-HLA-E mAb enhancement of cytotoxicity. Furthermore, tumor biopsies can be monitored before and during the course of therapy using these monospecific HLA-E mAbs.

## Notes

^†^ HLA antigens representing various alleles on the HLA-Ia beadset are listed on the One Lambda website under “Antibody Detection Products/LABScreen Single Antigen Products.” The single recombinant HLA-Ia in beadset (cat. #LS1A04, lot 007) includes 31 HLA-A, 50 HLA-B, and 16 HLA-Cw antigens, together with in-built control beads, coated with human IgG (considered positive control) or serum albumin (human or bovine) (negative control).

## References

[1] Lanier LL, and Phillips JH: Ontogeny of natural killer cells. Nature 1986;319:269–270 3484543 10.1038/319269b0

[2] Bezman NA, Kim CC, Sun JC, Min-Oo G, Hendricks DW, Kamimura Y, Best JA, Goldrath AW, and Lanier LL; Immunological genome project consortium: Molecular definition of natural killer cell identity and activation. Nat Immunol 2012;13:1000–1009 22902830 10.1038/ni.2395 PMC3572860

[3] Rajalingam R: The impact of HLA class I-specific killer cell immunoglobulin-like receptors on antibody-dependent natural killer cell-mediated cytotoxicity and organ allograft rejection. Front Immunol 2016;7:585 28066408 10.3389/fimmu.2016.00585 PMC5165035

[4] Rajalingam R: Overview of the killer cell immunoglobulin-like receptor system. Methods Mol Biol 2012;882:391–414 22665247 10.1007/978-1-61779-842-9_23

[5] Natarajan K, Dimasi N, Wang J, Margulies DH, and Mariuzza RA: MHC class I recognition by Ly49 natural killer cell receptors. Mol Immunol 2002;38:1023–1027 11955594 10.1016/s0161-5890(02)00031-7

[6] Moretta L, Bottino C, Pende D, Castriconi R, Mingari MC, and Moretta A: Surface NK receptors and their ligands on tumor cells. Semin Immunol 2006;18:151–158 16730454 10.1016/j.smim.2006.03.002

[7] Houchins JP, Yabe T, McSherry C, and Bach FH: DNA sequence analysis of NKG2, a family of related cDNA clones encoding type II integral membrane proteins on human natural killer cells. J Exp Med 1991;173:1017–1020 2007850 10.1084/jem.173.4.1017 PMC2190798

[8] Lazetic S, Chang C, Houchins JP, Lanier LL, and Phillips JH: Human natural killer cell receptors involved in MHC class I recognition are disulfide-linked heterodimers of CD94 and NKG2 subunits. J Immunol 1996;157:4741–4745 8943374

[9] Borrego F, Ulbrecht M, Weiss EH, Coligan JE, and Brooks AG: Recognition of human histocompatibility leukocyte antigen (HLA)-E complexed with HLA class I signal sequence-derived peptides by CD94/NKG2 confers protection from natural killer cell-mediated lysis. J Exp Med 1998;187:813–818 9480992 10.1084/jem.187.5.813 PMC2212178

[10] Braud VM, Allan DS, O'Callaghan CA, Söderström K, D'Andrea A, Ogg GS, Lazetic S, Young NT, Bell JI, Phillips JH, Lanier LL, and McMichael AJ: HLA-E binds to natural killer cell receptors CD94/NKG2A, B and C. Nature 1998;391:795–799 9486650 10.1038/35869

[11] Hayakawa Y, Kelly JM, Westwood JA, Darcy PK, Diefenbach A, Raulet D, and Smyth MJ: Cutting edge: Tumor rejection mediated by NKG2D receptor-ligand interaction is dependent upon perforin. J Immunol 2002;169:5377–5381 12421908 10.4049/jimmunol.169.10.5377

[12] Cerwenka A, and Lanier LL: Natural killers join the fight against cancer. Science 2018;359:1460–1461 29599226 10.1126/science.aat2184

[13] Groh V, Bahram S, Bauer S, Herman A, Beauchamp M, and Spies T: Cell stress regulated human major histocompatibility complex class I gene expressed in gastrointestinal epithelium. Proc Natl Acad Sci U S A 1996;93:12445–12450 8901601 10.1073/pnas.93.22.12445 PMC38011

[14] Groh V, Steinle A, Bauer S, and Spies T: Recognition of stress-induced MHC molecules by intestinal epithelial γδT cells. Science 1998;279:1737–1740 9497295 10.1126/science.279.5357.1737

[15] Groh V, Wu J, Yee C, and Spies T: Tumour-derived soluble MIC ligands impair expression of NKG2D and T-cell activation. Nature 2002;419:734–738 12384702 10.1038/nature01112

[16] Groh V, Rhinehart R, Randolph-Habecker J, Topp MS, Riddell SR, and Spies T: Co-stimulation of CD8 αβ T cells by NKG2D via engagement by MIC induced on virus-infected cells. Nat Immunol 2001;2:255–260 11224526 10.1038/85321

[17] Radosavljevic M, Cuillerier B, Wilson MJ, Clement O, Wicker S, Gilfillan S, Beck S, Trowsdale J, and Bahram S: A cluster of ten novel MHC class I related genes on human chromosome 6q24.2-q25.3. Genomics 2002;79:114–123 11827464 10.1006/geno.2001.6673

[18] Kaiser BK, Yim D, Chow IT, Gonzalez S, Dai Z, Mann HH, Strong RK, Groh V, and Spies T: Disulphide-isomerase-enabled shedding of tumour-associated NKG2D ligands. Nature 2007;447:482–486 17495932 10.1038/nature05768

[19] Ferrari de Andrade L, Tay RE, Pan D, Luoma AM, Ito Y, Badrinath S, Tsoucas D, Franz B, May KF Jr, Harvey CJ, Kobold S, Pyrdol JW, Yoon C, Yuan GC, Hodi FS, Dranoff G, and Wucherpfennig KW: Antibody-mediated inhibition of MICA and MICB shedding promotes NK cell-driven tumor immunity. Science 2018;359:1537–1542 29599246 10.1126/science.aao0505 PMC6626532

[20] Sheppard S, Ferry A, Guedes J, and Guerra N: The paradoxical role of NKG2D in cancer immunity. Front Immunol 2018;9:1808 30150983 10.3389/fimmu.2018.01808 PMC6099450

[21] Raulet DH: Roles of the NKG2D immunoreceptor and its ligands. Nat Rev Immunol 2003;3:781–790 14523385 10.1038/nri1199

[22] Zhang J, Basher F, and Wu JD: NKG2D ligands in tumor immunity: Two sides of a coin. Front Immunol 2015;6:97 25788898 10.3389/fimmu.2015.00097 PMC4349182

[23] Zhao Y, Chen N, Yu Y, Zhou L, Niu C, Liu Y, Tian H, Lv Z, Han F, and Cui J: Prognostic value of MICA/B in cancers: A systematic review and meta-analysis. Oncotarget 2017;8:96384–96395 29221214 10.18632/oncotarget.21466 PMC5707108

[24] Prajapati K, Perez C, Rojas LBP, Burke B, and Guevara-Patino JA: Functions of NKG2D in CD8(+) T cells: An opportunity for immunotherapy. Cell Mol Immunol 2018;15:470–479 29400704 10.1038/cmi.2017.161 PMC6068164

[25] Trembath AP, and Markiewicz MA: More than decoration: Roles for natural killer group 2 member D ligand expression by immune cells. Front Immunol 2018;9:231 29483917 10.3389/fimmu.2018.00231 PMC5816059

[26] Schmiedel D, and Mandelboim O: NKG2D ligands–critical targets for cancer immune escape and therapy. Front Immunol 2018;9:2040 30254634 10.3389/fimmu.2018.02040 PMC6141707

[27] Li P, Morris DL, Willcox BE, Steinle A, Spies T, and Strong RK: A complex structure of the activating immunoreceptor NKG2D and its MHC class I-like ligand MICA. Nat Immunol 2001;2:443–451 11323699 10.1038/87757

[28] Li P, McDermott G, and Strong RK: Crystal structures of RAE-1β and its complex with the activating immunoreceptor NKG2D. Immunity 2002;16:77–86 11825567 10.1016/s1074-7613(02)00258-3

[29] Li P, Willie ST, Bauer S, Morris DL, Spies T, and Strong RK: Crystal structure of the MHC class I homolog MIC-A, a gammadelta T cell ligand. Immunity 1999;10:577–584 10367903 10.1016/s1074-7613(00)80057-6

[30] McFarland BJ, Kortemme T, Yu SF, Baker D, and Strong RK: Symmetry recognizing asymmetry: Analysis of the interactions between the C-type lectin-like immunoreceptor NKG2D and MHC class I-like ligands. Structure 2003;11:411–422 12679019 10.1016/s0969-2126(03)00047-9

[31] Ravindranath MH, Zhu D, Pham T, Jucaud V, Hopfield J, Kawakita S, and Terasaki PI: Anti-HLA-E monoclonal antibodies reacting with HLA-la and lb alleles like IVIg as potential IVIg-immunomimetics: An evolving therapeutic concept. Clin Transpl 2013;2013:293–305 25095521

[32] Ortaldo JR, and Herberman RB: 4. Heterogeneity of natural killer cells. Annu Rev Immunol 1984;2:359–394 6399848 10.1146/annurev.iy.02.040184.002043

[33] Karre K, Ljunggren HG, Piontek G, and Kiessling R: Selective rejection of H-2 deficient lymphoma variants suggests alternative immune defense strategy. Nature 1986;319:675–678 3951539 10.1038/319675a0

[34] Liao NS, Bix M, Zijstra M, Jaenisch R, and Raulet D: MHC class I deficiency: Susceptibility to natural killer (NK) cells and impaired NK activity. Science 1991;253:199–202 1853205 10.1126/science.1853205

[35] Storkus WJ, Howell DN, Salter RD, Dawson JR, and Cresswell PJ: NK susceptibility varies inversely with target cell class I HLA antigen expression. J Immunol 1987;138:1657–1659 3819393

[36] Moretta A, Vitale M, Sivori S, Bottino C, Morelli L, Augugliaro R, Barbaresi M, Pende D, Ciccone E, Lopez-Botet M, and Moretta L: Human natural killer cell receptors for HLA-class I molecules: Evidence that the Kp43 (CD94) molecule functions as receptor for HLA-B alleles. J Exp Med 1994;180:545–555 8046333 10.1084/jem.180.2.545 PMC2191622

[37] Carretero M, Cantoni C, Bellón T, Bottino C, Biassoni R, Rodríguez A, Pérez-Villar JJ, Moretta L, Moretta A, and López-Botet M: The CD94 and NKG2-A C-type lectins covalently assemble to form a natural killer cell inhibitory receptor for HLA class I molecules. Eur J Immunol 1997;27:563–567 9045931 10.1002/eji.1830270230

[38] Mingari MC, Ponte M, Bertone S, Schiavetti F, Vitale C, Bellomo R, Moretta A, and Moretta L: HLA class I-specific inhibitory receptors in human T lymphocytes: Interleukin 15-induced expression of CD94/NKG2A in superantigen- or alloantigen-activated CD8^+^ T cells. Proc Natl Acad Sci U S A 1998;95:1172–1177 9448304 10.1073/pnas.95.3.1172 PMC18710

[39] Moretta A, Bottino C, Vitale M, Pende D, Biassoni R, Mingari MC, and Moretta L: Receptors for HLA-class I molecules in human natural killer cells. Annu Rev Immunol 1998;14:619–648 10.1146/annurev.immunol.14.1.619 8717527

[40] Braud V, Jones EY, and McMichael A: The human major histocompatibility complex class Ib molecule HLA-E binds signal sequence-derived peptides with primary anchor residues at positions 2 and 9. Eur J Immunol 1997;27:1164–1169 9174606 10.1002/eji.1830270517

[41] O'Callaghan CA: Natural killer cell surveillance of intracellular antigen processing pathways mediated by recognition of HLA-E and Qa-1b by CD94/NKG2 receptors. Microbes Infect 2000;2:371–380 10817639 10.1016/s1286-4579(00)00330-0

[42] Barnes PD, and Grundy JE: Down regulation of class I HLA heterodimer and beta 2-microglobulin on the surface of cells infected with cytomegalovirus. J Gen Virol 1992;73:2395–2403 1328494 10.1099/0022-1317-73-9-2395

[43] Fruh K, Ahn K, Djaballah H, Sempe P, Van Endert PM, Tampe R, Peterson PA, and Yang Y: A viral inhibitor of peptide transporters for antigen presentation. Nature 1995;375:415–418 7760936 10.1038/375415a0

[44] Ahn K, Meyer TH, Uebel S, Sempé P, Djaballah H, Yang Y, Peterson PA, Früh K, Tampé R: Molecular mechanism and species specificity of TAP inhibition by herpes simplex virus ICP47. EMBO J 1996;15:3247–3255 8670825 PMC451885

[45] Lee N, Llano M, Carretero M, Ishitani A, Navarro F, López-Botet M, and Geraghty DE: HLA-E is a primary ligand for the natural killer inhibitory receptor CD94/NKG2A. Proc Natl Acad Sci U S A 1998;95:5199–5204 9560253 10.1073/pnas.95.9.5199 PMC20238

[46] Brooks AG, Borrego F, Posch PE, Patamawenu A, Scorzelli CJ, Ulbrecht M, Weiss EH, and Coligan JE: Specific recognition of HLA-E, but not classical, HLA class I molecules by soluble CD94/NKG2A and NK cells. J Immunol 1999;162:305–313 9886400

[47] Ravindranath MH, Taniguchi M, Chen CW, Ozawa M, Kaneku H, El-Awar N, Cai J, Terasaki PI HLA-E monoclonal antibodies recognize shared peptide sequences on classical HLA class Ia: relevance to human natural HLA antibodies. Mol Immunol 2010;47:1121–1131 19944464 10.1016/j.molimm.2009.10.024

[48] Petrie EJ, Clements CS, Lin J, Sullivan LC, Johnson D, Huyton T, Heroux A, Hoare HL, Beddoe T, Reid HH, Wilce MC, Brooks AG, and Rossjohn J: CD94-NKG2A recognition of human leukocyte antigen (HLA)-E bound to an HLA class I leader sequence. J Exp Med 2008;205:725–735 18332182 10.1084/jem.20072525 PMC2275392

[49] Sullivan LC, Clements CS, Beddoe T, Johnson D, Hoare HL, Lin J, Huyton T, Hopkins EJ, Reid HH, Wilce MC, Kabat J, Borrego F, Coligan JE, Rossjohn J, and Brooks AG: The heterodimeric assembly of the CD94-NKG2 receptor family and implications for human leukocyte antigen-E recognition. Immunity 2007;27:900–911 18083576 10.1016/j.immuni.2007.10.013

[50] Hoare HL, Sullivan LC, Clements CS, Ely LK, Beddoe T, Henderson KN, Lin J, Reid HH, Brooks AG, and Rossjohn J: Subtle changes in peptide conformation profoundly affect recognition of the non-classical MHC class I molecule HLA-E by the CD94-NKG2 natural killer cell receptors. J Mol Biol 2008;377:1297–1303 18339401 10.1016/j.jmb.2008.01.098

[51] Hoare HL, Sullivan LC, Pietra G, Clements CS, Lee EJ, Ely LK, Beddoe T, Falco M, Kjer-Nielsen L, Re5id HH, McCluskey J, Moretta L, Rossjohn J, and Brooks AG: Structural basis for a major histocompatibility complex class Ib-restricted T cell response. Nat Immunol 2006;7:256–264 16474394 10.1038/ni1312

[52] Kaiser BK, Barahmand-Pour F, Paulsene W, Medley S, Geraghty DE and Strong RK: Interactions between NKG2x immunoreceptors and HLA-E ligands display overlapping affinities and thermodynamics. J Immunol 2005;174:2878–2884 15728498 10.4049/jimmunol.174.5.2878

[53] Vales-Gomez M, Reyburn HT, Erskine RA, Lopez-Botet M, and Strominger JL: Kinetics and peptide dependency of the binding of the inhibitory NK receptor CD94/NKG2-A and the activating receptor CD94/NKG2-C to HLA-E. EMBO J 1999;18:4250–4260 10428963 10.1093/emboj/18.15.4250 PMC1171501

[54] Ravindranath MH, Terasaki PI, Pham T, and Jucaud V: The monospecificity of novel anti-HLA-E monoclonal antibodies enables reliable immunodiagnosis, immunomodulation of HLA-E, and upregulation of CD8^+^ T lymphocytes. Monoclon Antib Immunodiagn Immunother 2015;34:135–153 26090591 10.1089/mab.2014.0096

[55] Sibilio L, Martayan A, Fraioli R, Lo Monaco E, Melucci E, Suchanek M, and Giacomini P: Biochemical characterization of monoclonal antibodies to HLA-E and HLA-G. Tissue Antigens 2003;62:356

[56] Lo Monaco E, Sibilio L, Melucci E, Tremante E, Suchànek M, Horejsi V, Martayan A, and Giacomini P: HLA-E: Strong association with beta2-microglobulin and surface expression in the absence of HLA class I signal sequence-derived peptides. J Immunol 2008;181:5442–5450 18832701 10.4049/jimmunol.181.8.5442

[57] Menier C, Saez B, Horejsi V, Martinozzi S, Krawice-Radanne I, Bruel S, Le Danff C, Reboul M, Hilgert I, Tableau M, Larrad ML, Pla M, Carosella D, and Rouas-Freiss N: Characterization of monoclonal antibodies recognizing HLA-G or HLA-E:newtools to analyze the expression of nonclassical HLA class Imolecules. Hum Immunol 2003;64:315–326 12590976 10.1016/s0198-8859(02)00821-2

[58] Lo Monaco E, Tremante E, Cifaldi L, Fruci D, and Giacomini P: HLA-E and the origin of immunogenic self-HLA epitopes. Mol Immunol 2010;47, 1660–1662 10.1016/j.molimm.2009.12.018 20096934

[59] Tremante E, Ginebri A, Lo Monaco E, Benassi B, Frascione P, Grammatico P, Cappellacci S, Catricala C, Arcelli D, Natali PG, Di Filippo F, Mottolese M, Visca P, Benevolo M, Giacomini P:ôA melanoma immune response signature including Human Leukocyte Antigen-E. Pigment Cell Melanoma Res 2014;27:103–112 24011128 10.1111/pcmr.12164

[60] Tremante E, Lo Monaco E, Ingegnere T, Sampaoli C, Fraioli R, and Giacomini P: Monoclonal antibodies to HLA-E bind epitopes carried by unfolded β2 m-free heavy chains. Eur J Immunol 2015;45:2356–2364 25982269 10.1002/eji.201545446

[61] Lee N, Goodlett DR, Ishitani A, Marquardt H, and Geraghty DE: HLA-E surface expression depends on binding of TAP-dependent peptides derived from certain HLA class I signal sequences. J Immunol 1998;160:4951–4960 9590243

[62] Ravindranath MH, Pham T, El-Awar N, Kaneku H, and Terasaki PI: Anti-HLA-E mAb 3D12 mimics MEM-E/02 in binding to HLA-B and HLA-C alleles: Web-tools validate the immunogenic epitopes of HLA-E recognized by the antibodies. Mol Immunol 2011;48:423–430 21145594 10.1016/j.molimm.2010.09.011

[63] Jucaud V, Ravindranath MH, and Terasaki PI: Conformational variants of the individual HLA-I antigens on Luminex single antigen beads used in monitoring HLA antibodies: Problems and solutions. Transplantation 2017;101:764–777 27495776 10.1097/TP.0000000000001420 PMC7228605

[64] Ravindranath MH, Terasaki PI, Pham T, Jucaud V, and Kawakita S: Therapeutic preparations of IVIg contain naturally occurring anti-HLA-E antibodies that react with HLA-Ia (HLA-A/-B/-Cw) alleles. Blood 2013;121:2013–2028 23305735 10.1182/blood-2012-08-447771

[65] Otten HG, Verhaar MC, Borst HP, van Eck M, van Ginkel WG, Hené RJ, and van Zuilen AD: The significance of pretransplant donor-specific antibodies reactive with intact or denatured human leucocyte antigen in kidney transplantation. Clin Exp Immunol 2013;173:536–543 23627692 10.1111/cei.12127 PMC3949641

[66] Oaks M, Michel K, Sulemanjee NZ, Thohan V, and Downey FX: Practical value of identifying antibodies to cryptic HLA epitopes in cardiac transplantation. J Heart Lung Transplant 2014;33:713–720 24661683 10.1016/j.healun.2014.02.013

[67] El Hilali F, Jucaud V, El Hilali H, Bhuiyan M, Mancuso A, LiuSullivan N, Elidrissi A, Mazouz H Profiles of Anti-HLA class I and II IgG antibodies in Moroccan IVIg determined by Luminex Multiplex Single antigen beads Immunoassay. Intl J Immunol 2017;5:53–65

[68] Visentin J, Guidicelli G, Bachelet T, Jacquelinet C, Audry B, Nong T, Dubois V, Moreau JF, Lee JH, Couzi L, Merville P, and Taupin JL: Denatured class I human leukocyte antigen antibodies in sensitized kidney recipients: Prevalence, relevance, and impact on organ allocation. Transplantation 2014;98:738–744 25289917 10.1097/TP.0000000000000229

[69] Visentin J, Marroc M, Guidicelli G, Bachelet T, Nong T, Moreau JF, Lee JH, Merville P, Couzi L, and Taupin JL: Clinical impact of preformed donor-specific denatured class I HLA antibodies after kidney transplantation. Clin Transplant 2015;29:393–402 25683727 10.1111/ctr.12529

[70] Visentin J, Guidicelli G, Nong T, Moreau JF, Merville P, Couzi L, Lee JH, and Taupin JL: Evaluation of the iBeads assay as a tool for identifying class I HLA antibodies. Hum Immunol 2015;76:651–656 26407911 10.1016/j.humimm.2015.09.012

[71] Sasaki T, Ravindranath MH, Terasaki PI, Freitas MC, Kawakita S, and Jucaud V: Gastric cancer progression may involve a shift in HLA-E profile from an intact heterodimer to β2-microglobulin-free monomer. Int J Cancer 2014;134:1558–1570 24105714 10.1002/ijc.28484

[72] Schnabl E, Stockinger H, Majdic O, Gaugitsch H, Lindley IJ, Maurer D, Hajek-Rosenmayr A, and Knapp W: Activated human T lymphocytes express MHC class I heavy chains not associated with beta 2-microglobulin. J Exp Med 1990;171:1431–1442 2139695 10.1084/jem.171.5.1431 PMC2187879

[73] Madrigal JA, Belich MP, Benjamin RJ, Little AM, Hildebrand WH, Mann DL, and Parham P: Molecular definition of a polymorphic antigen (LA45) of free HLA-A and -B heavy chains found on the surface of the activated B and T cells. J Exp Med 1991;174:1085–1095 1940790 10.1084/jem.174.5.1085 PMC2119017

[74] Matko J, Bushkin Y, Wei T, and Edidin M: Clustering of class I HLA molecules on the surfaces of activated and transformed human cells. J Immunol 1994;152:3353–3360 8144921

[75] Ravindranath MH, Terasaki PI, Pham T, Jucaud V, and Kawakita S: Suppression of blastogenesis and proliferation of activated CD4(+) T cells: Intravenous immunoglobulin (IVIg) versus novel anti-human leucocyte antigen (HLA)-E monoclonal antibodies mimicking HLA-I reactivity of IVIg. Clin Exp Immunol 2014;178:154–177 24889882 10.1111/cei.12391 PMC4360205

[76] van Esch EM, Tummers B, Baartmans V, Osse EM, Ter Haar N, Trietsch MD, Hellebrekers BW, Holleboom CA, Nagel HT, Tan LT, Fleuren GJ, van Poelgeest MI, van der Burg SH, and Jordanova ES: Alterations in classical and nonclassical HLA expression in recurrent and progressive HPV-induced usual vulvar intraepithelial neoplasia and implications for immunotherapy. Int J Cancer 2014;135:830–842 24415578 10.1002/ijc.28713

[77] Kren L, Slaby O, Muckova K, Lzicarova E, Sova M, Vybihal V, Svoboda T, Fadrus P, Lakomy R, Vanhara P, Krenova Z, Sterba J, Smrcka M, and Michalek J: Expression of immune-modulatory molecules HLA-G and HLA-E by tumor cells in glioblastomas: An unexpected prognostic significance? Neuropathology 2011;31:129–134 20667016 10.1111/j.1440-1789.2010.01149.x

[78] Bossard C, Bézieau S, Matysiak-Budnik T, Volteau C, Laboisse CL, Jotereau F, and Mosnier JF: HLA-E/β2 microglobulin overexpression in colorectal cancer is associated with recruitment of inhibitory immune cells and tumor progression. Int J Cancer 2012;131:855–863 21953582 10.1002/ijc.26453

[79] O'Callaghan CA, Tormo J, Willcox BE, Braud VM, Jakobsen BK, Stuart DI, McMichael AJ, Bell JI, and Jones EY: Structural features impose tight peptide binding specificity in the nonclassical MHC molecule HLA-E. Mol Cell 1998;1:531–541 9660937 10.1016/s1097-2765(00)80053-2

[80] Strong RK, Holmes MA, Li P, Braun L, Lee N, and Geraghty DE: HLA-E allelic variants. Correlating differential expression, peptide affinities, crystal structures, and thermal stabilities. J Biol Chem 2003;278:5082–5090 12411439 10.1074/jbc.M208268200

[81] Boyington JC, Motyka SA, Schuck P, Brooks AG, and Sun PD: Crystal structure of an NK cell immunoglobulin-like receptor in complex with its class I MHC ligand. Nature 2000;405:537–543 10850706 10.1038/35014520

[82] Pietra G, Romagnani C, Mazzarino P, Falco M, Millo E, Moretta A, Moretta L, and Mingari MC: HLA-E-restricted recognition of cytomegalovirus-derived peptides by human CD8^+^ cytolytic T lymphocytes. Proc Natl Acad Sci U S A 2003;100:10896–10901 12960383 10.1073/pnas.1834449100 PMC196899

[83] Nattermann J, Nischalke HD, Hofmeister V, Ahlenstiel G, Zimmermann H, Leifeld L, Weiss EH, Sauerbruch T, and Spengler U: The HLA-A2 restricted T cell epitope HCV core 35–44 stabilizes HLA-E expression and inhibits cytolysis mediated by natural killer cells. Am J Pathol 2005;166:443–453 15681828 10.1016/S0002-9440(10)62267-5 PMC1602324

[84] Wooden SL, Kalb SR, Cotter RJ, and Soloski MJ: Cutting edge: HLA-E binds a peptide derived from the ATP-binding cassette transporter multidrug resistance-associated protein 7 and inhibits NK cell-mediated lysis. J Immunol 2005;175:1383–1387 16034073 10.4049/jimmunol.175.3.1383

[85] Nattermann J, Nischalke HD, Hofmeister V, Kupfer B, Ahlenstiel G, Feldmann G, Rockstroh J, Weiss EH, Sauerbruch T, and Spengler U: HIV-1 infection leads to increased HLA-E expression resulting in impaired function of natural killer cells. Antivir Ther 2005;10:95–107 15751767 10.1177/135965350501000107

[86] Derré L, Corvaisier M, Charreau B, Moreau A, Godefroy E, Moreau-Aubry A, Jotereau F, and Gervois N: Expression and release of HLA-E by melanoma cells and melanocytes: Potential impact on the response of cytotoxic effector cells. J Immunol 2006;177:3100–3107 16920947 10.4049/jimmunol.177.5.3100

[87] Gonçalves AS, Oliveira JP, Oliveira CF, Silva TA, Mendonça EF, Wastowski IJ, and Batista AC: Relevance of HLA-G, HLA-E and IL-10 expression in lip carcinogenesis. Hum Immunol 2016;77:785–790 26723902 10.1016/j.humimm.2015.12.001

[88] Silva TG, Crispim JC, Miranda FA, Hassumi MK, de Mello JM, Simões RT, Souto F, Soares EG, Donadi EA, and Soares CP: Expression of the nonclassical HLA-G and HLA-E molecules in laryngeal lesions as biomarkers of tumor invasiveness. Histol Histopathol 2011;26:1487–1497 21972088 10.14670/HH-26.1487

[89] Djajadiningrat RS, Horenblas S, Heideman DA, Sanders J, de Jong J, and Jordanova ES: Classic and nonclassic HLA class I expression in penile cancer and relation to HPV status and clinical outcome. J Urol 2015;193:1245–1251 25463996 10.1016/j.juro.2014.11.057

[90] Mittelbronn M, Simon P, Löffler C, Capper D, Bunz B, Harter P, Schlaszus H, Schleich A, Tabatabai G, Goeppert B, Meyermann R, Weller M, and Wischhusen J: Elevated HLA-E levels in human glioblastomas but not in grade I to III astrocytomas correlate with infiltrating CD8^+^ cells. J Neuroimmunol 2007;189:50–58 17675252 10.1016/j.jneuroim.2007.07.002

[91] Kren L, Muckova K, Lzicarova E, Sova M, Vybihal V, Svoboda T, Fadrus P, Smrcka M, Slaby O, Lakomy R, Vanhara P, Krenova Z, and Michalek J: Production of immune-modulatory nonclassical molecules HLA-G and HLA-E by tumor infiltrating ameboid microglia/macrophages in glioblastomas: A role in innate immunity? J Neuroimmunol 2010;220:131–135 20167379 10.1016/j.jneuroim.2010.01.014

[92] Costa Arantes DA, Gonçalves AS, Jham BC, Duarte ECB, de Paula ÉC, de Paula HM, Mendonça EF, and Batista AC: Evaluation of HLA-G, HLA-E, and PD-L1 proteins in oral osteosarcomas. Oral Surg Oral Med Oral Pathol Oral Radiol 2017;123:e188–e196 28159587 10.1016/j.oooo.2016.12.002

[93] Mosconi C, Arantes DAC, Gonçalves AS, Alencar RCG, Oliveira JC, Silva TA, Mendonça EF, and Batista AC: Immunohistochemical investigations on the expression of programmed cell death ligand 1, human leukocyte antigens G and E, and granzyme B in intraoral mucoepidermoid carcinoma. Arch Oral Biol 2017;83:55–62 28711734 10.1016/j.archoralbio.2017.07.004

[94] Reimers MS, Engels CC, Putter H, Morreau H, Liefers GJ, van de Velde CJ, and Kuppen PJ: Prognostic value of HLA class I, HLA-E, HLA-G and Tregs in rectal cancer: A retrospective cohort study. BMC Cancer 2014;14:486 24997850 10.1186/1471-2407-14-486 PMC4094545

[95] Benevolo M, Mottolese M, Tremante E, Rollo F, Diodoro MG, Ercolani C, Sperduti I, Lo Monaco E, Cosimelli M, and Giacomini P: High expression of HLA-E in colorectal carcinoma is associated with a favorable prognosis. J Transl Med 2011;9:184 22032294 10.1186/1479-5876-9-184 PMC3219584

[96] Zhen ZJ, Ling JY, Cai Y, Luo WB, and He YJ: Impact of HLA-E gene polymorphism on HLA-E expression in tumor cells and prognosis in patients with stage III colorectal cancer. Med Oncol 2013;30:482 23377987 10.1007/s12032-013-0482-2

[97] Zeestraten EC, Reimers MS, Saadatmand S, Goossens-Beumer IJ, Dekker JW, Liefers GJ, van den Elsen PJ, van de Velde CJ, and Kuppen PJ: Combined analysis of HLA class I, HLA-E and HLA-G predicts prognosis in colon cancer patients. Br J Cancer 2014;110:459–468 24196788 10.1038/bjc.2013.696 PMC3899753

[98] Guo ZY, Lv YG, Wang L, Shi SJ, Yang F, Zheng GX, Wen WH, and Yang AG: Predictive value of HLA-G and HLA-E in the prognosis of colorectal cancer patients. Cell Immunol 2015;293:10–16 25461612 10.1016/j.cellimm.2014.10.003

[99] Huang R, Zhang D, Li F, Xiao Z, Wu M, Shi D, Xiang P, and Bao Z: Loss of Fas expression and high expression of HLA-E promoting the immune escape of early colorectal cancer cells. Oncol Lett 2017;13:3379–3386 28521443 10.3892/ol.2017.5891 PMC5431327

[100] Chen A, Shen Y, Xia M, Xu L, Pan N, Yin Y, Miao F, Shen C, Xie W, and Zhang J: Expression of the nonclassical HLA class I and MICA/B molecules in human hepatocellular carcinoma. Neoplasma 2011;58:371–376 21744989 10.4149/neo_2011_05_371

[101] Talebian Yazdi M, van Riet S, van Schadewijk A, Fiocco M, van Hall T, Taube C, Hiemstra PS, and van der Burg SH: The favorable prognostic effect of stromal CD8^+^ tumor-infiltrating T cells is restrained by the expression of HLA-E in non-small cell lung carcinoma. Oncotarget 2016;7:3477–3488 26658106 10.18632/oncotarget.6506 PMC4823121

[102] de Kruijf EM, Sajet A, van Nes JG, Natanov R, Putter H, Smit VT, Liefers GJ, van den Elsen PJ, van de Velde CJ, and Kuppen PJ: HLA-E and HLA-G expression in classical HLA class I-negative tumors is of prognostic value for clinical outcome of early breast cancer patients. J Immunol 2010;185:7452–7459 21057081 10.4049/jimmunol.1002629

[103] da Silva GB, Silva TG, Duarte RA, Neto NL, Carrara HH, Donadi EA, Gonçalves MA, Soares EG, and Soares CP: Expression of the classical and nonclassical HLA molecules in breast cancer. Int J Breast Cancer 2013;2013:250435 24363939 10.1155/2013/250435 PMC3864140

[104] Gooden M, Lampen M, Jordanova ES, Leffers N, Trimbos JB, van der Burg SH, Nijman H, and van Hall T: HLA-E expression by gynecological cancers restrains tumor-infiltrating CD8^+^ T lymphocytes. Proc Natl Acad Sci U S A 2011;108:10656–10661 21670276 10.1073/pnas.1100354108 PMC3127933

[105] Gonçalves MA, Le Discorde M, Simões RT, Rabreau M, Soares EG, Donadi EA, and Carosella ED: Classical and non-classical HLA molecules and p16 (INK4a) expression in precursors lesions and invasive cervical cancer. Eur J Obstet Gynecol Reprod Biol 2008;141:70–74 18692948 10.1016/j.ejogrb.2008.06.010

[106] Spaans VM, Peters AA, Fleuren GJ, and Jordanova ES: HLA-E expression in cervical adenocarcinomas: Association with improved long-term survival. J Transl Med 2012;10:184 22947189 10.1186/1479-5876-10-184 PMC3480912

[107] Ferns DM, Heeren AM, Samuels S, Bleeker MCG, de Gruijl TD, Kenter GG, and Jordanova ES: Classical and non-classical HLA class I aberrations in primary cervical squamous- and adenocarcinomas and paired lymph node metastases. J Immunother Cancer 2016;4:78 27895918 10.1186/s40425-016-0184-3 PMC5109766

[108] Andersson E, Poschke I, Villabona L, Carlson JW, Lundqvist A, Kiessling R, Seliger B, and Masucci GV: Non-classical HLA-class I expression in serous ovarian carcinoma: Correlation with the HLA-genotype, tumor infiltrating immune cells and prognosis. Oncoimmunology 2015;5:e1052213 26942060 10.1080/2162402X.2015.1052213 PMC4760332

[109] Zheng H, Lu R, Xie S, Wen X, Wang H, Gao X, and Guo L: Human leukocyte antigen-E alleles and expression in patients with serous ovarian cancer. Cancer Sci 2015;106:522–528 25711417 10.1111/cas.12641 PMC4452152

[110] Hanak L, Slaby O, Lauerova L, Kren L, Nenutil R, and Michalek J: Expression pattern of HLA class I antigens in renal cell carcinoma and primary cell line cultures: Methodological implications for immunotherapy. Med Sci Monit 2009;15:CR638–CR643 19946235

[111] Kren L, Valkovsky I, Dolezel J, Capak I, Pacik D, Poprach A, Lakomy R, Redova M, Fabian P, and Krenova Z: Slaby O HLA-G and HLA-E specific mRNAs connote opposite prognostic significance in renal cell carcinoma. Diagn Pathol 2012;7:58 22640987 10.1186/1746-1596-7-58 PMC3408319

[112] Zanetti BR, Carvalho-Galano DF, Feitosa NL, Hassumi-Fukasawa MK, Miranda-Camargo FA, Maciel LM, Ribeiro-Silva A, and Soares EG: Differential expression of immune-modulatory molecule HLA-E in non-neoplastic and neoplastic lesions of the thyroid. Int J Immunopathol Pharmacol 2013;26:889–896 24355224 10.1177/039463201302600407

[113] Kren L, Fabian P, Slaby O, Janikova A, Soucek O, Sterba J, Krenova Z, Michalek J, and Kral Z: Multifunctional immune-modulatory protein HLA-E identified in classical Hodgkin lymphoma: Possible implications. Pathol Res Pract 2012;208:45–49 22177730 10.1016/j.prp.2011.11.004

[114] Sensi M, Pietra G, Molla A, Nicolini G, Vegetti C, Bersani I, Millo E, Weiss E, Moretta L, Mingari MC, and Anichini A: Peptides with dual binding specificity for HLA-A2 and HLA-E are encoded by alternatively spliced isoforms of the antioxidant enzyme peroxiredoxin 5. Int Immunol 2009;21:257–268 19181932 10.1093/intimm/dxn141

[115] Marín R, Ruiz-Cabello F, Pedrinaci S, Méndez R, Jiménez P, Geraghty DE, and Garrido F: Analysis of HLA-E expression in human tumors. Immunogenetics 2003;54:767–775 12618909 10.1007/s00251-002-0526-9

[116] Wischhusen J, Friese MA, Mittelbronn M, Meyermann R, and Weller M: HLA-E protects glioma cells from NKG2D-mediated immune responses *in vitro*: Implications for immune escape *in vivo*. J Neuropathol Exp Neurol 2005;64:523–528 15977644 10.1093/jnen/64.6.523

[117] Wolpert F, Roth P, Lamszus K, Tabatabai G, Weller M, and Eisele G: HLA-E contributes to an immune-inhibitory phenotype of glioblastoma stem-like cells. J Neuroimmunol 2012;250:27–34 22688424 10.1016/j.jneuroim.2012.05.010

[118] Morandi F, Pozzi S, Carlini B, Amoroso L, Pistoia V, and Corrias MV: Soluble HLA-G and HLA-E levels in bone marrow plasma samples are related to disease stage in neuroblastoma patients. J Immunol Res 2016;2016:7465741 27610393 10.1155/2016/7465741 PMC5004009

[119] McWilliams EM, Mele JM, Cheney C, Timmerman EA, Fiazuddin F, Strattan EJ, Mo X, Byrd JC, Muthusamy N, and Awan FT: Therapeutic CD94/NKG2A blockade improves natural killer cell dysfunction in chronic lymphocytic leukemia. Oncoimmunology 2016;5:e1226720 27853650 10.1080/2162402X.2016.1226720 PMC5087289

[120] Wagner B, da Silva Nardi F, Schramm S, Kraemer T, Celik AA, Dürig J, Horn PA, Dührsen U, Nückel H, and Rebmann V: HLA-E allelic genotype correlates with HLA-E plasma levels and predicts early progression in chronic lymphocytic leukemia. Cancer 2017;123:814–823 27859015 10.1002/cncr.30427

[121] Zhen Z, Guo X, Liao R, Yang K, Ye L, and You Z: Involvement of IL-10 and TGF-β in HLA-E-mediated neuroblastoma migration and invasion. Oncotarget 2016;7:44340–44349 27322426 10.18632/oncotarget.10041 PMC5190101

[122] Özgül Özdemir RB, Özdemir AT, Oltulu F, Kurt K, Yiğittürk G, and Kırmaz C: A comparison of cancer stem cell markers and nonclassical major histocompatibility complex antigens in colorectal tumor and noncancerous tissues. Ann Diagn Pathol 2016;25:60–63 27806848 10.1016/j.anndiagpath.2016.09.012

[123] Stangl S, Gross C, Pockley AG, Asea AA, and Multhoff G: Influence of Hsp70 and HLA-E on the killing of leukemic blasts by cytokine/Hsp70 peptide-activated human natural killer (NK) cells. Cell Stress Chaperones 2008;13:221–230 18759005 10.1007/s12192-007-0008-y PMC2673894

[124] Allard M, Oger R, Vignard V, Percier JM, Fregni G, Périer A, Caignard A, Charreau B, Bernardeau K, Khammari A, Dréno B, and Gervois N: Serum soluble HLA-E in melanoma: A new potential immune-related marker in cancer. PLoS One 2011;6:e21118 21712991 10.1371/journal.pone.0021118 PMC3119680

[125] Levy EM, Bianchini M, Von Euw EM, Barrio MM, Bravo AI, Furman D, Domenichini E, Macagno C, Pinsky V, Zucchini C, Valvassori L, and Mordoh J: Human leukocyte antigen-E protein is overexpressed in primary human colorectal cancer. Int J Oncol 2008;32:633–641 18292941

[126] Levy EM, Sycz G, Arriaga JM, Barrio MM, von Euw EM, Morales SB, González M, Mordoh J, and Bianchini M: Cetuximab-mediated cellular cytotoxicity is inhibited by HLA-E membrane expression in colon cancer cells. Innate Immun 2009;15:91–100 19318419 10.1177/1753425908101404

